# Comparative physiology and morphology of BLA-projecting NBM/SI cholinergic neurons in mouse and macaque

**DOI:** 10.21203/rs.3.rs-4824445/v1

**Published:** 2024-08-02

**Authors:** Feng Luo, Li Jiang, Niraj S. Desai, Li Bai, Gabrielle V. Watkins, Mark A. G. Eldridge, Anya Plotnikova, Arya Mohanty, Alex C. Cummins, Bruno B. Averbeck, David A. Talmage, Lorna W. Role

**Affiliations:** 1 Section on Circuits, Synapses, and Molecular Signaling, National Institute of Neurological Disorders and Stroke, National Institutes of Health, Bethesda, 20892, MD, USA; 2 Section on Genetics of Neuronal Signaling, National Institute of Neurological Disorders and Stroke, National Institutes of Health, Bethesda, 20892, MD, USA; 3 Laboratory of Neuropsychology, National Institute of Mental Health, Bethesda, 20892, MD, USA

**Keywords:** cholinergic, nonhuman primate, mouse, basal forebrain, morpho-electric physiology

## Abstract

Cholinergic projection neurons of the nucleus basalis and substantia innominata (NBM/SI) densely innervate the basolateral amygdala (BLA) and have been shown to contribute to the encoding of fundamental and life-threatening experiences. Given the vital importance of these circuits in the acquisition and retention of memories that are essential for survival in a changing environment, it is not surprising that the basic anatomical organization of the NBM/SI is well conserved across animal classes as diverse as teleost and mammal. What is not known is the extent to which the physiology and morphology of NBM/SI neurons have also been conserved. To address this issue, we made patch-clamp recordings from NBM/SI neurons in *ex vivo* slices of two widely divergent mammalian species, mouse and rhesus macaque, focusing our efforts on cholinergic neurons that project to the BLA. We then reconstructed most of these recorded neurons *post hoc* to characterize neuronal morphology. We found that rhesus macaque BLA-projecting cholinergic neurons were both more intrinsically excitable and less morphologically compact than their mouse homologs. Combining measurements of 18 physiological features and 13 morphological features, we illustrate the extent of the separation. Although macaque and mouse neurons both exhibited considerable within-group diversity and overlapped with each other on multiple individual metrics, a combined morpho-electric analysis demonstrates that they form two distinct neuronal classes. Given the shared purpose of the circuits in which these neurons participate, this finding raises questions about (and offers constraints on) how these distinct classes result in similar behavior.

## INTRODUCTION

The basal forebrain cholinergic system is among the most antique parts of the brain, being present in vertebrates as evolutionarily distant as teleosts and primates (Semba, 2004). Among mammals, its organization follows a similar anatomical plan across the rostral-caudal axis that includes the ventral pallidum, medial septum, diagonal band, substantia innominata (SI), and nucleus basalis of Meynert (NBM) (Mesulam et al., 1983b; Woolf, 1991). These nuclei send long-range projections to cortex, hippocampus, and amygdala, and are thought to subserve similar fundamental functions across species: participating in attention and sensory perception, avoidance of aversive or life-threatening situations, and reinforcement of appetitive behaviors. Overall, the cholinergic system for many species is implicated in embedding important experiences in memory (Picciotto et al., 2012; Ballinger et al., 2016; Knox and Keller, 2016; Ananth et al., 2023).

Although the similarities across evolution are striking, there are also notable differences in the structure and composition of the basal forebrain in distinct species. For example, the volume of the nucleus basalis is an order of magnitude larger in nonhuman primates than it is in rodents (Semba, 2004), and cholinergic neurons have been estimated to account for as much as 50–90% of the NBM neurons in primates (Mesulam et al., 1983a; Mesulam and Geula, 1988; Bañuelos et al., 2023) but only 5–20% in rodents (Gritti et al., 2006; Zaborszky et al., 2012). This mix of similarities and differences is both perplexing and intriguing. It raises questions both about how different species produce equivalent behaviors and about how their responses might differ.

These questions are especially important with regard to the cholinergic neurons of the NBM/SI that send long-range projections to the basolateral amygdala (BLA). These neurons are part of the survival circuits in the brain that are conserved across mammalian species (LeDoux, 2012, 2022; McGaugh, 2018; Diehl et al., 2019). NBM/SI cholinergic neurons send a particularly dense innervation to BLA, and the resulting circuit is engaged in both appetitive (Tye, 2018; Crouse et al., 2020) and aversive (Jiang et al., 2016; Rajebhosale et al., 2024) memory encoding. Cued appetitive or aversive stimuli excite NBM/SI cholinergic neurons and lead to increased release of acetylcholine in the BLA (Crouse et al., 2020; Rajebhosale et al., 2024). Acquisition of fear-associated and reward-associated memories is enhanced through increased BLA principal neuron activity, mediated at least in part by cholinergic signaling. Optogenetic stimulation of cholinergic terminals in the BLA reinforces cue-reward learning (Crouse et al., 2020) and extends the durability of fear memory against extinction in mice (Jiang et al., 2016). Likewise, in both rodents and primates, lesions of basal forebrain cholinergic projection neurons, or chemogenetic and/or pharmacological interference with cholinergic signaling, disrupt multiple types of memory acquisition and retention in these species (Ridley et al., 1999; Turchi et al., 2005; Easton et al., 2011; Melamed et al., 2017; Rajebhosale et al., 2024).

In this study, we approach the questions using a *bottom-up* approach: we characterize the building blocks of cholinergic circuits – namely, the intrinsic electrical properties and morphology of individual cholinergic neurons – with the idea that this starts us on an understanding of circuit computations as a whole. We identified BLA-projecting neurons in NBM/SI in two very different mammalian species, mouse and monkey (rhesus macaque), by retrograde transport of fluorescently-labeled microbeads. We determined their cholinergic phenotype by immunohistochemical labeling (monkey) and/or genetic expression of a choline acetyltransferase (ChAT) transgene (mouse) and characterized their intrinsic electrical properties using whole-cell patch-clamp recordings. We reconstructed these physiologically characterized neurons using neurobiotin processing to trace the shapes of their proximal neuritic arbors. We then used the resulting physiological and morphological measurements, together with standard techniques for dimensionality reduction, to define quantitively, how mouse and monkey neurons in this well-defined set are similar and how they are different at the cellular level.

## MATERIALS AND METHODS

All animal use was conducted under an animal study protocol approved by the NINDS or NIMH Animal Care and Use Committees (ACUC) and conformed to the Institute of Medicine Guide for the Care and Use of Laboratory Animals.

### Mouse surgery and tissue preparation

Twenty-seven ChAT-tau-GFP mice (Grybko et al., 2011) were used in this study (18 males and 9 females, 1.5 – 3.5 months, Table 1). For microbead injections, mice were anesthetized with isoflurane and positioned in a stereotaxic apparatus (David Kopf Instruments). Eye ointment was applied to prevent the cornea from drying out, and Meloxicam SR (2 mg/ml) was injected subcutaneously to alleviate pain. Using aseptic techniques, a cut along the anterior-posterior axis was made to expose the skull. After craniotomy holes were drilled, a 26-gauge microsyringe (Hamilton) was used to deliver microbeads (FluoSpheres^™^ Carboxylate-Modified Microspheres, red F8793, Invitrogen; 200 – 300 nl) into the BLA (anterior/posterior −1.1 mm from bregma; medial/lateral ± 3.25 mm from bregma; dorsal/ventral −4.15 mm from dura) bilaterally. After injections, the scalp was repositioned using Vetbond (3M) tissue adhesive and lidocaine cream was applied topically. Animals were returned to the home cage with a heating pad for recovery.

Five to seven days after surgery, animals were anesthetized with ketamine/xylazine (100 and 10 mg/kg, respectively, delivered i.p.) and transcardially perfused with an ice-cold cutting solution (in mM: 230 sucrose, 2.5 KCl, 10 MgSO_4_, 0.5 CaCl_2_, 1.25 NaH_2_PO_4_, 26 NaHCO_3_, 10 glucose and 1.5 sodium pyruvate, pH 7.4, osmolarity 300–310 mOsm) oxygenated with carbogen (95% O_2_/5% CO_2_). After decapitation, the brain was quickly removed from the skull and immersed for several minutes in ice-cold oxygenated cutting solution.

Coronal brain slices (250 or 300 μm) were prepared with a Leica VT1200S vibratome and then transferred to a holding chamber containing artificial cerebrospinal fluid (aCSF) solution (in mM: 126 NaCl, 2.5 KCl, 1.25 NaH_2_PO_4_, 26 NaHCO_3_, 2 CaCl_2_, 2 MgCl_2_ and 10 glucose, pH 7.4, osmolarity 300–310 mOsm) oxygenated with carbogen. Slices were equilibrated at room temperature for at least 1 hour prior to transfer to a recording chamber perfused with oxygenated aCSF at 31 ± 0.5 °C.

### Monkey craniotomy and injection

From 13 total monkeys (*Macaca mulatta*), data on BLA projecting cholinergic neurons were obtained from nine adults, including seven males (9.8 ± 2.0 years old; ranging from 7.2 to 12.8 years) and two females (6.9 and 16.9 years old; see Table 1 for demographic details and data summary).

Surgeries were carried out in a veterinary operating facility using aseptic technique. Structural MRIs were used to guide the brain injections (Saunders et al., 1990). Animals were sedated with ketamine hydrochloride (10 mg/kg), and anesthesia was maintained with isoflurane. Body temperature, heart rate, blood pressure, SpO_2_ and expired CO_2_ were monitored throughout. Stereotaxic injection coordinates were derived from pre-operative structural MRIs (Saunders et al., 1990; Walbridge et al., 2006). The procedure has been described in detail in previous studies (Fredericks et al., 2020). Microbeads (FluoSpheres^™^ Carboxylate-Modified Microspheres, blue F8781 or red F8793, Invitrogen) were sterile filtered (0.45 μm filter unit, Merck Millipore) and injected in a volume of 20 μL per site, at 1.0 μL/min. Three injection sites were placed 2 – 2.5 mm apart in the dorsoventral plane at each of two anterior-posterior sites 1.5 – 2.0 mm apart, for a total injection volume of 120 μL across 6 sites. In a subset of experiments, AAV.PHP.Eb S9E27::dTom NLS-dTom developed by Dr. Fishell and colleagues (Furlanis et al., 2024) was also used. Accordingly, in some animals, two additional injection sites above anterior NBM/SI were placed 4.0 – 5.0 mm apart in the medio-lateral plane for a volume of 10 μL per site, at 1.0 μL/min. However, in all cases, the identification of neurons as cholinergic in monkey was based solely on *post hoc* staining with ChAT antibody.

### Monkey tissue harvest

On the morning of tissue harvest, each animal was sedated with ketamine hydrochloride (10 mg/kg i.m) and perfused (70–80 ml/min) with ice-cold slicing buffer (in mM: 90 sucrose, 80 NaCl, 3.5 KCl, 24 NaHCO_3_, 1.25 NaH_2_PO_4_, 4.5 MgCl_2_, 0.5 CaCl_2_ and 10 glucose, pH 7.4, osmolarity 290–300 mOsm) oxygenated with carbogen, until the lungs were white and no blood came out of the right atrium. The brain was rapidly removed (~5 min) from the skull and submerged in ice-cold carbogen-bubbled aCSF. The brain was separated into two hemispheres, and then blocked in the coronal plane at two levels. The first cut was through the most rostral part of the temporal lobe, and the second cut was performed about 13 mm caudal to the first cut at the level of rostral hippocampus. The isolated tissue was blocked to ~10 mm wide × 10 mm high × 6 mm thick to contain only striatum, the basal forebrain, and the BLA. The tissue block was placed in ice-cold oxygenated perfusion solution and transported to the electrophysical laboratory for slicing. Coronal slices at a thickness of 250–300 μm were obtained using a Leica VT1200S vibratome in an ice-cold cutting solution optimized for aged tissues (Ting et al., 2018). The solution had an osmolarity of ~300 mOsm, was bubbled continuously with carbogen, and contained (in mM): 92 N-methyl-D-glucamine, 2.5 KCl, 1.25 NaH_2_PO_4_, 30 NaHCO_3_, 20 HEPES, 25 glucose, 2 thiourea, 5 Na-ascorbate, 3 Na-pyruvate, 0.5 CaCl_2_·2H_2_O, and 10 MgSO_4_·7H_2_O (titrated to pH 7.3–7.4 with concentrated hydrochloric acid). Slices were then transferred to a holding chamber that contained carbogen-bubbled solution (in mM): 92 NaCl, 2.5 KCl, 1.25 NaH_2_PO_4_, 30 NaHCO_3_, 20 HEPES, 25 glucose, 2 thiourea, 5 Na-ascorbate, 3 Na-pyruvate, 2 CaCl_2_·2H_2_O, and 2 MgSO_4_·7H_2_O; pH 7.4; osmolarity 300–310 mOsm. Slices were equilibrated at room temperature for at least 1 hour prior to recording.

### Electrophysiology

Whole-cell patch-clamp recordings were made from neurons in brain slices containing NBM/SI from either mouse or monkey using identical procedures. Slices were perfused continuously with oxygenated aCSF at a rate of 1–2 mL/min and a temperature of 31.0 ± 0.5 °C. The NBM/SI region was located by using the anterior commissure, internal capsule, optic tract and/or striatum as landmarks (locations of all recorded neurons were confirmed *post hoc* by relocalization of neurobiotin-filled cells and assessed for the presence or absence of ChAT immunoreactivity, as delineated below). Neurons were selected for recording based on their location and the presence of microbeads from the previous BLA injection. Labeled neurons were visualized with a high-resolution camera on an upright microscope fitted with differential interference contrast optics and a fluorescence microscopy illumination system (Slice Pro 6000, Scientifica). Recordings were made with PatchStar manipulators (Scientifica) using borosilicate glass electrodes (4–6 MΩ) pulled on a laser-based puller (P-2000, Sutter) and filled with intracellular solution containing the following (in mM): 125 K-gluconate, 10 KCl, 1 MgCl_2_, 10 HEPES, 4 Mg-ATP 0.3 Na_2_-GTP, 7 phosphocreatine, 0.2% neurobiotin, pH 7.3 corrected with KOH, osmolarity 290–295 mOsm). All recordings were amplified (10x) and low-pass filtered (4 kHz) with Multiclamp 700B amplifiers (Molecular Devices), digitized using either a Digidata 1550b (Molecular Devices) or a National Instruments PCIe-6353 board, and acquired at 10 kHz with either pClamp 11 software (Molecular Devices) or custom scripts written in MATLAB (The Mathworks). Recordings were analyzed offline using custom MATLAB code (see below Electrophysiology analysis).

Once a recording was in whole-cell (current-clamp) mode and stabilized following equilibration with the intracellular solution, passive and active membrane properties of the neurons were measured using a family of current steps (500 ms duration, −60 to 200 pA amplitude). For these recordings, the baseline membrane potential was adjusted to −65 mV and current steps were separated by at least 10 sec to allow for recovery. Recordings were accepted for off-line analysis only if access resistance (<25 MΩ) was stable to within <20%, offset current in whole-cell configuration remained within 10% of its initial value, and the resting membrane potential was ≤−40 mV. Liquid junction potentials (−14 mV) were not corrected.

### Immunohistochemistry and imaging

After patch-clamp recordings, sections were fixed in 4% paraformaldehyde in 0.1 M PBS (pH 7.4) overnight at 4 °C and transferred to phosphate-buffered saline (PBS) for storage until processing.

For visualization of the neurobiotin-labelled cells in mouse, free-floating sections were rinsed in PBS (3 × 10 mins) at room temperature before being incubated in streptavidin dye conjugate (Cy5, 1:1000, Sigma) with 2% Triton X-100 over night at 4 °C. The slices were washed in PBS (3 × 10 mins) and mounted for imaging with DAPI fluoromount. In control experiments, we contrasted mouse morphological features obtained with standard fixation protocol against those obtained with Clear, Unobstructed Brain Imaging Cocktails (CUBIC): there were no significant differences in measures of proximal arbor metrics (convex hull, complexity, numbers, lengths, etc.; data not shown). Thus, we grouped data from both the standard fixation protocol and CUBIC for analysis.

CUBIC was used for visualization of the neurobiotin-labelled macaque neurons. Free-floating sections were rinsed in PBS (3 × 60 mins) and incubated in CUBIC solution A (25% N,N,N’N’ – tetrakis (2-hydroxypropyl) ethylendiamine, 25% urea, 15% Triton-X in Milli-Q water) on a shaker for 1–2 days at room temperature. The procedures for immunohistochemical detection of ChAT were as follows. After being rinsed in PBS (3 × 60 mins), the sections were blocked in a PBS solution containing 10% normal donkey serum and 2% TritonX-100 for 16–24 hours on a shaker at 4 °C. The blocking solution was used as the diluent in all subsequent antibody solutions. Brain sections were incubated with a goat polyclonal primary antibody (1:250; AB144P, Millipore Sigma) against ChAT, shaking at 4 °C for six nights. Following primary antibody incubation, sections were washed in dilution buffer (3 × 60 mins) at RT (20–25 °C) and incubated in Alexa-Fluor donkey anti-goat 488 (1:1000) and streptavidin dye conjugate (Cy5, 1:1000, Sigma) for 16–24 hours at 4 °C. Sections were rinsed in PBS (2 × 60 mins) and transferred to CUBIC solution B (50% sucrose, 25% urea, 10% Triethanolamine, 0.1% Triton-X 100 in Milli-Q water) on a shaker overnight at RT. All incubations were conducted in darkness. Sections were mounted on Superfrost Plus^™^ glass slides and cover-slipped with CUBIC solution B. Coverslips were then sealed with clear nail polish (Electron Microscopy Sciences) and stored in a slide box at 4 °C until imaging within 2–4 hours.

Z-stacks of brain slices were imaged on a slide scanner (VS200, Olympus) for cell relocalization, and individual neurobiotin-relocated NBM/SI neurons were imaged on a confocal microscope (LSM800, Zeiss) using a 20x objective. Stacks were collected at a 1–2 μm slice interval, stepping through the entire soma and all visible processes of the neuron. All scans containing z-stack images were saved as CZI files (Olympus) for subsequent quantitative analysis.

### Electrophysiology analysis

Eighteen features were extracted from the responses to currents steps using custom code (available at https://doi.org/10.5281/zenodo.10975327) written in MATLAB (see Extended Data Fig. 2-1A). (1) Resting membrane potential (mV), measured in the absence of a current injection. (2) Sag potential (mV), measured in response to a −60 pA step, equal to the difference between the steady-state potential and the minimum potential. (3) Input resistance (MΩ), measured by the response to a −20 pA step. (4) Membrane time constant (τ, ms), measured by the relaxation to a −20 pA step. (5) Rheobase (pA), the minimum current step of 500 ms duration needed to elicit an action potential. (6) Spike threshold (mV), measured from the first action potential of the rheobase current step (“first action potential”), and defined as the potential at which dV/dt crosses 10 mV/ms. (7) Spike amplitude (mV), measured from the first action potential, and defined as the difference between the tip of the action potential and the spike threshold. (8) Spike width (ms), measured from the first action potential, and defined as the width at half maximum (halfway between threshold and tip). (9) Spike latency (ms), measured at rheobase current, and defined as the time difference between the start of the step and the threshold crossing of the first spike. (10) Upstroke (mV/ms), the maximum value of dV/dt on the upstroke of the first action potential. (11) Downstroke (mV/ms), the minimum value of dV/dt on the downstroke of the first action potential. (12) Afterhyperpolarization potential (AHP) amplitude (mV), measured after the first action potential, and defined as the difference between threshold and the minimum potential 100 ms later. (13) AHP latency (ms), the time after spike threshold is crossed by the first action potential and the AHP minimum. (14) AHP width (ms), the time difference at half maximum of the first AHP. (15) f-I slope (Hz/pA), the slope of the initial linear section of the f-I curve. (16) Max firing rate (Hz), the maximum firing rate produced by a current step between 0 and 200 pA, across the entire 500 ms duration. (17) Adaptation index (dimensionless), for the maximal current step, the number of spikes elicited in the second half of the step divided by the number elicited in the first half. (18) Coefficient of variation (CV, dimensionless) of interspike intervals, measured from the maximal current step.

### Cell reconstruction and morphological analysis

Confocal images of relocalized cells were imported to Imaris 9 software (Oxford Instruments) for filament tracing. Images were processed to remove background noise, and automated detection was used for morphological reconstruction of all neurites. The autogenerated filaments were fine-tuned manually to eliminate mis-detected branches and/or to add back undetected branches. Thirteen morphological features were quantified (Extended Data Fig. 2-1C and D) using these reconstructions.

The thirteen features were defined as follows. (1) Total process area (μm^2^), the summed surface area of all processes extending from the soma. This measurement excludes the area of the soma itself. (2) Total process length (μm) is the summed length of all processes extending from the soma. (3) Number of primary processes is the number of processes emerging directly from the soma. (4) Total process length normalized is the total process length divided by the number of primary processes. (5) Number of branch points is the total number of branching points in the whole neuritic tree. (6) Branch point normalized is the number of branch points divided by the number of primary processes. (7) Number of first order processes is the number of branches emerging from a primary process. (8) Number of second order processes is the number of branches emerging from a first order process. (9) Root angle (rad) was measured as in Bird and Cuntz (2019) using the TREES Toolbox (www.treestoolbox.org) (Bird and Cuntz, 2019). The root angle at a particular point is defined as the angle between the tangent line at that point and a line directly connecting the point to the soma (see Extended Data Fig. 2-1C). We measured the root angles at all points along the whole tree and calculated their average. (10) Centripetal bias (κ, dimensionless) was estimated by fitting the distribution of root angles to the 3-D von Mises distribution. The centripetal bias is roughly equivalent to the inverse of the distribution’s variance (1/σ^2). Thus, the centripetal bias is large for narrow, soma-oriented neuritic trees (e.g., dentate gyrus) and small for complicated, meandering trees (e.g., Purkinje cells) (Bird and Cuntz, 2019).

The convex hull is the minimal convex polyhedron that encloses all points of the whole arbor (see Extended Data Fig.2-1D). Convex Hull XTension (Oxford Instruments) was used to calculate the convex hull for each reconstruction. From this convex hull, three measurements were taken: (11) convex hull area (μm^2^), (12) convex hull volume (μm^3^), and (13) convex hull sphericity (dimensionless). Sphericity is defined as the ratio of the area if the entire convex hull volume were confined to a sphere and the measured convex hull area. A sphere is the most compact shape possible, and so the ratio will differ from (be smaller than) 1 depending on how non-spherical the convex hull shape is.

In addition to these 13 morphological measurements, which were our principal measures, we also calculated Sholl intersections in 3D (sphere) datasets with Imaris Filament Sholl Analysis. The script detects the filament starting point and calculates the intersections along the filament segments every 10 μm from the starting point.

### Statistical analysis

Statistical analyses were performed using MATLAB and its Statistics and Machine Learning Toolbox. Averages are represented as mean ± SD.

Each of the 18 physiological features extracted from the responses to current steps were compared between groups using a non-parametric Wilcoxon rank-sum test. A p-value less than 0.05 was taken to indicate significance. The same was true of the 13 morphological features.

To visualize group differences from many distinct features in two dimensions, we used the Uniform Manifold Approximation and Projection (UMAP) algorithm (McInnes et al., 2018). For all physiological recordings and/or morphological reconstructions used in each comparison, measured features were rendered dimensionless and of unit variance by z-score normalization across the data set. A principal component analysis was run to reduce dimensionality and minimize the effects of noise. Principal components that accounted for >1% of variance were retained. These were mapped onto two UMAP dimensions using a MATLAB implementation of the UMAP algorithm (see MATLAB Central File Exchange at https://www.mathworks.com/matlabcentral/fileexchange/71902 by Meehan and others).

Linear discriminant analyses (LDA) were performed using MATLAB function *fitcdiscr*.

## RESULTS

### Electrophysiology in the mouse: cholinergic versus non-cholinergic BLA projecting NBM/SI neurons

We first examined the electrophysiological properties of BLA-projecting NBM/SI neurons using whole-cell patch-clamp recordings in mouse brain slices (see complete workflow; Fig. 1). One week before each recording session, red fluorospheres (“microbeads”) were injected bilaterally into the BLA of ChAT-tau-GFP mice (Fig. 1A). The beads were taken up by axonal terminals in the BLA and transported retrogradely to projection areas, including NBM/SI. This allowed us to identify BLA-projecting NBM/SI neurons in brain slices by checking for red fluorescence (Fig. 1B, “Beads”). ChAT-tau-GFP mice express the tau-GFP fusion protein under the control of a ChAT promotor such that green fluorescence is expressed throughout the cholinergic neurons and their arbors (Fig. 1B, “GFP”). Therefore, we were able to identify both BLA-projecting cholinergic (red + green) and BLA-projecting non-cholinergic (red only) neurons in our recordings. We verified our neuron identification in two ways. First, immediately after slice preparation, before whole-cell patch-clamp recordings, we checked that the bead injection site was in BLA (Fig. 1B, micrograph at left; Extended Data Fig. 1-1). Second, slices were fixed and processed for confocal imaging after the recording session. Neurons that had been filled with neurobiotin during the whole-cell recordings could be relocalized by staining the neurobiotin with streptavidin (Fig. 1C). Not only did this post-recording procedure allow us to double-check for red (beads-labeled) and green (ChAT+) fluorescence but it allowed us to make morphological reconstructions of physiologically characterized neurons (as described in the next section).

In total, 48 BLA-projecting NBM/SI cholinergic neurons and 46 BLA-projecting non-cholinergic neurons were recorded in 56 brain slices from 27 animals (18 males; 9 females, Table 1). Cell relocalization indicated that 89% of the recorded neurons were located between bregma −0.46 mm and −0.94 mm, covering the full rostral-caudal axis of the NBM/SI (Extended Data Fig. 1-2).

Intrinsic electrophysiological properties were characterized by injecting a family of current steps (500 ms duration) with amplitudes between −60 and +200 pA. To standardize measurements, recordings were made from a baseline potential of −65 mV, maintained by a small offset current (offset currents had to be less than −100 pA to pass quality control). Sample traces at rheobase (Fig. 2A, left) and at maximum current injection (Fig. 2A, right) are shown for two typical BLA-projecting NBM/SI neurons (cholinergic: teal; non cholinergic: grey). The responses to the current steps were used to extract 18 electrophysiological features representing both subthreshold (e.g., resting input resistance and sag potential) and suprathreshold intrinsic properties (e.g., spike threshold and subsequent active currents; Extended Data Fig. 2-1A). Average phase plots constructed from the rheobase current step were also used to illustrate differences in action potential kinetics (Fig. 2B and Extended Data Figs. 2-1B). The results are shown in Figs. 2 and Extended Data Fig. 2-2.

Cholinergic and non-cholinergic BLA-projecting neurons within the region of the NBM/SI differed electrophysiologically in all but three features (Figs. 2D, Extended Data Figs. 2-2). Overall, non-cholinergic neurons were much more excitable than cholinergic ones, with a maximum firing rate almost six times larger (cholinergic 7.3 ± 4.3 Hz, non-cholinergic 39.0 ± 24.9 Hz, p<0.0001). This difference was driven both by changes in spike generation (spike threshold: cholinergic −33 ± 4.6 mV, non-cholinergic −37.1 ± 6.3 mV, p < 0.001; rheobase current: cholinergic 64.3 ± 41.2 pA, non-cholinergic 42.2 ± 33.2 pA, p<0.001) and by changes in afterhyperpolarization (AHP amplitude: cholinergic 28.9 ± 8.3, non-cholinergic 12.4 ± 6.1 mV, p < 0.0001; AHP width: cholinergic 177.9 ± 89.3 ms, non-cholinergic 85.0 ± 118.6 ms, p < 0.0001) with concomitant effects on spike frequency adaptation (adaptation index: cholinergic 0.83 ± 0.56, non-cholinergic 0.64 ± 0.29, p < 0.05). Passive electrophysiological properties, such as resting membrane potential (cholinergic −54.4 ± 8.4 mV, non-cholinergic −58.7 ± 8.2 mV, p<0.05) and membrane time constant tau (cholinergic 34.6 ± 14.3, non-cholinergic 22.3 ± 9.8 ms, p<0.0001), also exhibited significant differences.

Although there was considerable variability in each feature assayed within the cholinergic and non-cholinergic groups and considerable overlap between them in many features, the differences taken as a whole were so strong that unbiased, principal component analyses clearly separated cholinergic from non-cholinergic BLA projecting, NBM/SI neurons. We demonstrated this by reducing dimensionality by running the 18 physiological features through a principal components analysis (PCA), discarding PCA components that accounted for <1% of the variance. We displayed the remaining components in two dimensions using the universal manifold approximation and projection (UMAP) algorithm (McInnes et al., 2018). The resulting UMAP plot (Fig. 2C) from this unsupervised classification show almost complete separation between the BLA-projecting cholinergic and non-cholinergic neurons within the NBM/SI; only two neurons fall on the “wrong” side of the boundary.

### Morphology in the mouse: cholinergic versus non-cholinergic BLA projecting NBM/SI neurons

BLA-projecting neurons were filled with neurobiotin during recordings to allow us to reconstruct and quantify the proximal neuritic arbors of recorded neurons. Mouse BLA-projecting NBM/SI neurons, whether cholinergic or non-cholinergic, showed considerable morphological diversity in their proximal arbors (Fig. 3, cholinergic; Extended Data Fig. 3-1, non-cholinergic). Across the rostral-caudal axis, most neurons were multipolar in shape. The proximal arbors of some spanned several hundreds of microns from the soma, while others appeared limited to within ~100 μm of the soma boundary. As in our electrophysiological analysis, BLA-projecting neurons were identified by the presence of red microbeads and divided into cholinergic and non-cholinergic categories by the presence or absence of ChAT expression (ChAT tau-GFP, Figs. 4A and 4B).

We used a total of 13 measured features to characterize neuritic morphology, including total process length, number of branch points, convex hull area, and convex hull sphericity (Extended Data Figs. 2-1 C and D). In contrast to the electrophysiological differences between neighboring cholinergic and non-cholinergic BLA projecting NBM/SI neurons, we found major similarities with respect to proximal neuritic morphology (Figs. 4 and Extended Data Fig. 4-1). Three of the 13 features assayed were statistically significantly different (Fig. 4D): cholinergic neurons had more branch points (cholinergic 8.1 ± 5.4, non-cholinergic 6.6 ± 5.4, p<0.05), a smaller total proximal process area (cholinergic 4.6 ± 2.7 × 10^3^ μm^2^, non-cholinergic 6.6 ± 3.6 × 10^3^ μm^2^, p<0.05), and a smaller centripetal bias (cholinergic 12.9 ± 14.9, non-cholinergic 15.2 ± 10.1, p<0.05).

On a standard Sholl analysis of intersections as a function of distance from the soma, cholinergic and non-cholinergic neurons were also not significantly different (Extended Data Fig. 4-1A). Dimensionality reduction via PCA and subsequent display via UMAP likewise indicated that proximal neuritic morphology was similar between the two types of mouse BLA projecting neurons (Fig. 4C).

We also used linear discriminant analysis (LDA) on both the electrophysiological features and the morphological features. LDA is a supervised classification scheme that attempts to find linear combinations of features that best separate distinct groups, which are specified beforehand. In this case, the specified groups were BLA-projecting cholinergic neurons and BLA-projecting non-cholinergic neurons. Given the clean separation between these groups on the unsupervised electrophysiological UMAP plot (Fig. 2C), supervised LDA revealed a clear separation (Fig. 5A). The feature with the highest weight in the LDA vector was AHP amplitude (Fig. 5B). By contrast, even supervised LDA failed to separate cholinergic and non-cholinergic neurons morphologically (Fig. 5C and D). Indeed, the distribution was equivalent to the LDA plots that resulted when feature values were randomly shuffled (Fig. 5C inset).

Together, the data from this part of our study indicate that among BLA-projecting NBM/SI neurons, there are strong differences in the electrophysiological characteristics of cholinergic and neighboring non cholinergic neurons, with the latter having multiple features consistent with a higher level of excitability. Interestingly, there is also a disconnect between physiological and morphological properties with only the former distinguishing cholinergic NBM/SI neurons from non-cholinergic neighbors.

### Electrophysiology of BLA projecting NBM/SI cholinergic neurons: mouse versus macaque

Previous reports emphasizing how the cholinergic system is conserved between species (Semba, 2004) led us to ask which cellular properties of BLA-projecting NBM/SI cholinergic neurons might be shared and which might differ between mouse and macaque. To answer this question, we focused on a definable class of basal forebrain cholinergic neurons, comparing only BLA-projecting, NBM/SI cholinergic neurons. We developed a workflow to characterize the electrophysiological and morphological properties of BLA-projecting NBM/SI neurons in the macaque that paralleled our studies in the mouse (Fig. 6 and Extended Data Fig. 6-1). With an MRI-guided surgical procedure, we injected fluorescent microbeads into macaque BLA to target the basal forebrain neurons that project to the BLA (see methods for procedures for stereotaxic injection; Fig. 6A, Extended Data Fig. 6-1A). After allowing at least six weeks for microbeads to move in a retrograde direction to the NBM/SI, the monkeys were euthanized, and brain tissue was removed. The tissue from a given animal was blocked into a small piece containing basal forebrain for slice collection and incubation (Extended Data Fig. 6-1B and C). After verifying the presence of microbeads in BLA (Fig. 6C and Extended Data Fig. 6-2), bead-labelled NBM/SI neurons were targeted for whole-cell patch-clamp recording (Fig. 6B & Extended Data Fig. 6-1D). These neurons were later relocalized based on coordinates, neurobiotin, and then confirmed as positive by ChAT immunolabeling (Fig. 6C).

In total, we successfully relocalized 52 BLA-projecting cholinergic neurons and 11 BLA-projecting non-cholinergic neurons from 9 macaques. The approximate bregma location of all relocalized neurons is shown in Extended Data Fig. 6-3. Most neurons were found at bregma positions between −4.95 to −7.65 mm. This range is comparable to the bregma range we used in mouse (−0.46 to −0.94 mm) based on local anatomical landmarks and covered the major portion of the macaque NBM/SI. In macaque NBM/SI, the density of cholinergic neurons is thought to be much greater than in comparable regions of mouse (Mesulam et al., 1983a). Because of the relatively low number of BLA-projecting, non-cholinergic neurons in our monkey samples compared with mouse, we focused our analyses on just the cholinergic neurons identified as BLA-projecting when comparing the two species.

Forty-six of the 52 relocalized macaque cholinergic neurons obtained from 38 brain slices passed quality control for inclusion in the study (see Methods). As in our mouse studies, the properties were characterized using a family of current steps (500 ms duration, −60 to +200 pA amplitude) from a baseline potential of −65 mV. Sample traces from representative macaque and mouse neurons are shown in Fig. 7A.

We found significant differences between macaque and mouse BLA-projecting cholinergic neurons in intrinsic excitability, with macaque neurons being more excitable. The maximum firing rate was three times larger (macaque 26.3 ± 23.1 Hz, mouse 7.3 ± 4.3 Hz, p < 0.0001), the rheobase current was only a third as large (macaque 23.3 ± 16.5, mouse 64.3 ± 41.2 pA, p < 0.0001), and the slope of the f-I curve was twice as large (macaque 0.13 ± 0.10, mouse 0.06 ± 0.03, p<0.0001). The higher maximal firing rate of macaque neurons resulted from a combination of changes in spike generation (spike threshold: macaque −39.7 ± 5.9 mV, mouse −33.0 ± 4.6 mV, p<0.0001) and in afterhyperpolarization properties (AHP amplitude: macaque 19.1 ± 8.8 mV, mouse 28.9 ± 8.3 mV, p<0.0001; AHP width: macaque 106.6 ± 71.9 ms, mouse 177.9 ± 89.3 ms, p<0.0001). Macaque neurons also differed from mouse neurons in basic spike shape (spike width: macaque 1.32 ± 0.45 ms, mouse 1.15 ± 0.43 ms, p<0.01), especially evident in the average phase plots (Figs. 7B and Extended Data Fig. 7-1B). In all, 15 of the 18 electrophysiological features were in significantly different between macaque and mouse (Figs. 7D, Extended Data Fig. 7-1A and C).

As before, we used dimensionality reduction with PCA to investigate how distinct the two groups were from each other. The resulting two-dimensional UMAP plot is given in Fig. 7C. It exhibits a strong separation between groups.

### Morphology of BLA projecting, NBM/SI cholinergic neurons: mouse versus macaque

Next, we compared the morphological features of the proximal neuritic arbor of BLA-projecting NBM/SI cholinergic neurons in macaque to those in mouse. Reconstructions of all relocalized macaque cholinergic neurons are shown in Fig. 8, with the neurons arrayed along the rostral-caudal axis. As in the mouse, the proximal arbors of macaque BLA-projecting cholinergic neurons were morphologically diverse, with all neurons appearing to be multipolar and relatively simple.

We measured 13 morphological features from macaque neurons (Figs. 9D and Extended Data Fig. 9-1B) and compared these to the same measurements from mouse neurons. Although many morphological features were similar (Extended Data Fig. 9-1A), five of the 13 measured parameters exhibited significant differences. Most notably, those related to convex hull were different (convex hull volume: macaque 2.19 ± 1.63 × 10^3^ μm^3^, mouse 0.94 ± 0.77 × 10^3^ μm^3^, p<0.0001; convex hull area: macaque 1.15 ± 0.59 × 10^5^ μm^2^, mouse 0.83 ± 0.45 × 10^5^ μm^2^, p<0.05; convex hull sphericity: macaque 0.65 ± 0.08, mouse 0.51 ± 0.09, p<0.0001). Two representative images of BLA-projecting NBM/SI cholinergic neurons with fitted convex hulls are shown in Fig. 9A and B. In well characterized neurons, convex hull is used as a representation of the maximal expanse of the dendritic arbor (Bird and Cuntz, 2019). The larger convex hull volume and area indicate that macaque neurons occupy more three-dimensional physical space than the comparable mouse neurons (Extended Data Figs. 9-2 and 9-3), whereas the larger sphericity indicates that macaque cholinergic neurons are fuller whereas mouse neurons are flatter.

Using PCA to reduce dimensionality and UMAP to visualize significant PCA vectors in two dimensions, we obtained the UMAP plot of Fig. 9D. There was a tendency for macaque neurons to cluster away from mouse neurons (Fig. 9C), but the effect of clustering based on morphology alone was weak – compared, for example, to the electrophysiological UMAP of Fig. 7C.

Finally, we combined the electrophysiological and morphological measures for an integrated morpho-electric features analysis and asked how well this combination distinguished between BLA-projecting cholinergic neurons from the NBM/SI of macaque and mouse. The results are given in Fig. 10. In these analyses, we limited ourselves to samples for which we had electrophysiological recordings that had passed all quality control and that were successfully relocated and confirmed as BLA projecting and cholinergic (46 macaque neurons and 27 mouse neurons). First, we tried unsupervised classification as before, by running numbers through a PCA, retaining PCA vectors that captured at least 1% of the variance, and then visualizing the PCA results in two dimensions using the UMAP method (Fig. 10A). Second, we tried supervised classification using LDA (Fig. 10B), where macaque and mouse neurons were explicitly categorized. The features with the highest weight in the LDA vector analysis differentiating macaque from mouse were convex hull in morphology and rheobase, AHP width, and maximum firing rate in physiology (Fig. 10C).

## DISCUSSION

The primary purpose of this study was to assess the similarities and differences between mouse and macaque basal forebrain cholinergic projection neurons. To make the comparison as meaningful as possible, we focused on a subset of neurons located within the NBM/SI that project to the BLA. These neurons have a function in fear learning and memory that is conserved across evolutionary time (Johansen et al., 2011; Dal Monte et al., 2015; Gore et al., 2015; Sah et al., 2020; Murray and Fellows, 2022). We assessed 18 passive and active electrophysiological features and 13 morphological features in each of the recorded neurons. In total, 30 mice were surgically back-labeled with fluorospheres, and 122 BLA-projecting neurons were sampled. For comparison, 13 macaques were examined yielding 83 BLA-projecting neurons. Between 70–80% of the back-labeled BLA-projecting neurons (mouse 107; macaque 57) passed initial quality control and were processed for relocation based on neurobiotin filling, with 80–90% successfully relocated. In total, we obtained complete analyses of 94 BLA projecting neurons (48 cholinergic and 46 noncholinergic for electrophysiology; 31 cholinergic and 44 noncholinergic for morphology) from 27 mice (56 slices) and 46 BLA-projecting cholinergic neurons from 9 macaques (38 slices).

We built two complete data sets. One data set allowed a within-species (mouse) comparison of BLA-projecting NBM/SI neurons, comparing those that were positive for ChAT with those that were not (48 versus 46, respectively). The other data set provided for a between-species comparison (mouse versus macaque) of BLA-projecting NBM/SI neurons, all of which were cholinergic. Although there was a considerable diversity both within and between species, our analyses of these two groups revealed important distinctions in both cases. Broadly speaking, mouse BLA-projecting cholinergic neurons are less excitable than mouse BLA-projecting non-cholinergic neurons, and macaque BLA-projecting NBM/SI cholinergic neurons are more excitable than mouse BLA-projecting NBM/SI cholinergic neurons. In addition, the soma-proximal arbors of macaque BLA-projecting NBM/SI cholinergic neurons tend to have more branch points and occupy larger three-dimensional space than those of mouse.

### Comparison with previous studies

The physiological differences we found between BLA-projecting cholinergic and non-cholinergic neurons in the mouse NBM/SI mirrored those of previous studies that examined the basal forebrain using less strict criteria. Those studies were done in guinea pig (Griffith, 1988; Alonso et al., 1996), rat (Markram and Segal, 1990; Bengtson and Osborne, 2000) and mouse (Hedrick and Waters, 2010; López-Hernández et al., 2017); in no case did they attempt to identify projection targets. Despite the variety of approaches used to identify and characterize these populations, the most striking and common property of basal forebrain cholinergic neurons is their overall sluggish excitability, with lower spiking rates, more rapid accommodation, and stronger AHP currents, compared with non-cholinergic neurons. The overall lower spike frequencies of cholinergic neurons have also been demonstrated using *in vivo* extracellular recordings in mouse (Hangya et al., 2015).

To the best of our knowledge, this is the first study in macaque characterizing basal forebrain cholinergic projection neurons in detail using both patch-clamp physiology and morphometric assays. Prior *in vivo* electrophysiology studies of BFCNs with extracellular recording have shown a mean spontaneous firing rate of 25 Hz in SI neurons from rhesus monkeys (Wilson and Rolls, 1990), 20–30 Hz in medial basal forebrain including MS and DB neurons (Ledbetter et al., 2016), and 40–50 Hz in lateral basal forebrain including VP neurons (Ledbetter et al., 2016). The firing rate of SI neurons is close to the median value of our macaque cholinergic NBM/SI dataset. However, extracellular studies do not assess whether the recorded neurons are cholinergic, potentially complicating these comparisons with our dataset.

### Potential mechanisms underlying observed physiological differences

The most obvious source of differences in electrophysiological profiles are variations in the underlying channel encoding genes, their levels of expression, and/or the cellular distribution of channel proteins. The vast number of channel encoding genes, their complex regulatory motifs, and their varying contributions to different aspects of excitability preclude narrowing in on a single candidate gene. Nevertheless, it is known that the broad family of K+ channels (including the various leak, voltage- and calcium-gated K+ conductances) are important determinants of neuronal excitability, contributing to the latency, duration and fidelity of spiking with increasing depolarizing steps, as well as to the time course of repolarization and adaptation (Sah and Sah, 1996; Betancourt and Colom, 2000; Enyedi and Czirjak, 2010). A survey of transcriptomic cell types in the mouse nervous system reveals that cholinergic neurons in the pallidum are relatively enriched in their expression of KCNC2 voltage-gated potassium channels in comparison to noncholinergic neurons in the same area (Zeisel et al., 2018), an observation that is at least consistent with our within-species cholinergic versus non cholinergic comparison. Clearly, additional cell-specific transcriptomic experiments are needed to gather in-depth information on the expression patterns of the key conductances in BLA-projecting cholinergic neurons in mouse and macaque. Patch-seq, combining electrophysiological recordings with single-cell transcriptome profiling in the same cells (Lipovsek et al., 2021; Chiou et al., 2023; Yao et al., 2023), would be especially useful.

### Physiological consequences

The physiological differences between mouse and macaque neurons reported here are striking. What makes them remarkable is that in this study we restricted our attention to the BLA-projecting cholinergic neurons of NBM/SI. One might think – and we thought at the outset – that such a well-defined class would have well-defined and conserved properties. Previous studies that compared the intrinsic physiology of similarly well-defined classes of neurons in mouse and primate (human) neocortex found that physiological properties were indeed largely conserved (Kalmbach et al., 2018, 2021). But we found that this was not true for this cholinergic neuronal class. Leaving morphology aside, physiology alone cleanly separated mouse from macaque neurons (Fig. 7C).

In retrospect, we should not have been surprised because biological systems generally do not require uniformity to produce uniform results. Degeneracy – the idea that biological systems, formed through the complexities of evolution, find multiple solutions to common problems – is an established concept. In neuroscience, it has been made most concrete by work on how different combinations of ion channels produce the same neuronal firing patterns (Prinz et al., 2004; Haddad and Marder, 2018; Seenivasan and Narayanan, 2022). Inspired by immunology studies, degeneracy has been suggested as a “first principle” for understanding neural organization generally (Tononi et al., 1999; Edelman and Gally, 2001).

Individual cholinergic neurons show striking physiological differences. How might they, even so, mediate equivalent behavior? There are multiple possibilities. One set of possibilities is upstream of the NBM/SI. Inhibitory and excitatory inputs to the NBM/SI could compensate for the lesser excitability of the mouse neurons if the inhibition they received was sparser or weaker, if the excitation was more distributed or stronger, if the fluctuations of one or the other were slightly different. A different set of possibilities is downstream of NBM/SI. The cholinergic fibers that NBM/SI send to BLA are notoriously expansive, with long lengths and multiple branch points. Branch point failures, multiple release sites and pre and post synaptic sites of action by acetylcholine in the BLA are all real possibilities (Unal et al., 2015; Jiang et al., 2016). Although there are many possibilities, most of these are experimentally tractable. One can measure excitatory and inhibitory currents in NBM/SI neurons using patch-clamp recordings, mark PSD95 and gephyrin to count synapses and their distribution (Bensussen et al., 2020), combined with the latest sensors to assess the temporal and spatial profile of acetylcholine release when axons are activated (Sethuramanujam et al., 2021; Zhong et al., unpublished observation). Finally, given that we have built a combined data set of physiology and morphological properties, this is an ideal case for computational methods to provide explanatory power.

## Extended Data

**Fig. 1–1:**
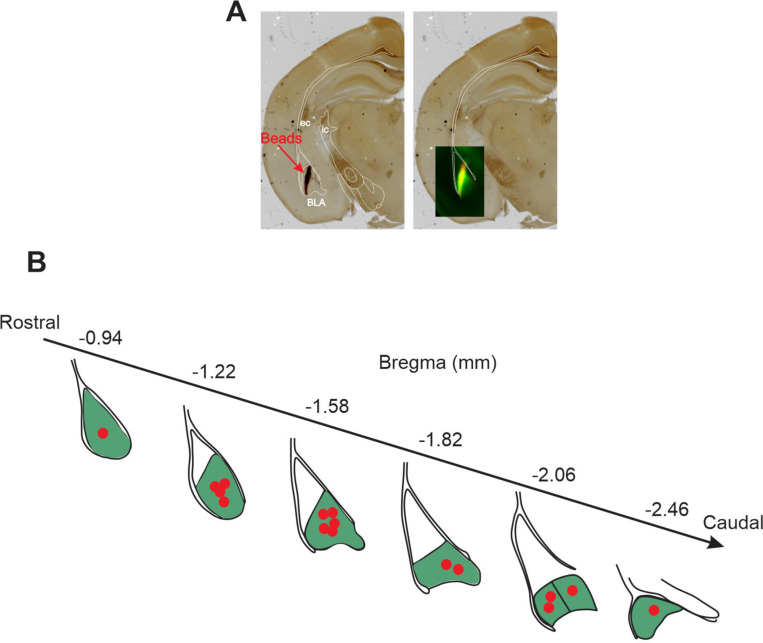
Reconstruction of the microbead injection sites in mouse BLA for retrograde labeling of BLA-projecting neurons. **A**. Representative slices showing major anatomical landmarks demarcating the BLA (green) and beads injection site (red). **B**. Beads target identification on slices in each animal. From 27 animals used for recordings, 16 animals were successfully scanned for site of beads injection, post recordings. BLA along the bregma is marked with Green. Each red dot represents the injection site from one animal.

**Fig. 1–2:**
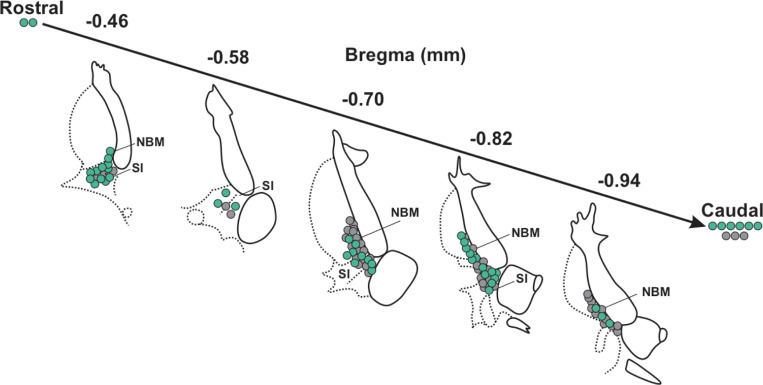
Approximate location of recorded, and relocalized BLA-projecting cholinergic and non-cholinergic neurons along bregma in mouse. Schematic diagram of coronal views along bregma of NBM/SI regions (Left shown here) in which we found BLA projecting neurons. These cells comprise the sample for electrophysiological and morphological features. Approximate locations of all BLA-projecting, NBM/SI cholinergic neurons (n= 48) are shown in teal; the locations of BLA-projecting, non-cholinergic neurons (n= 46) are shown in grey.

**Fig. 2–1:**
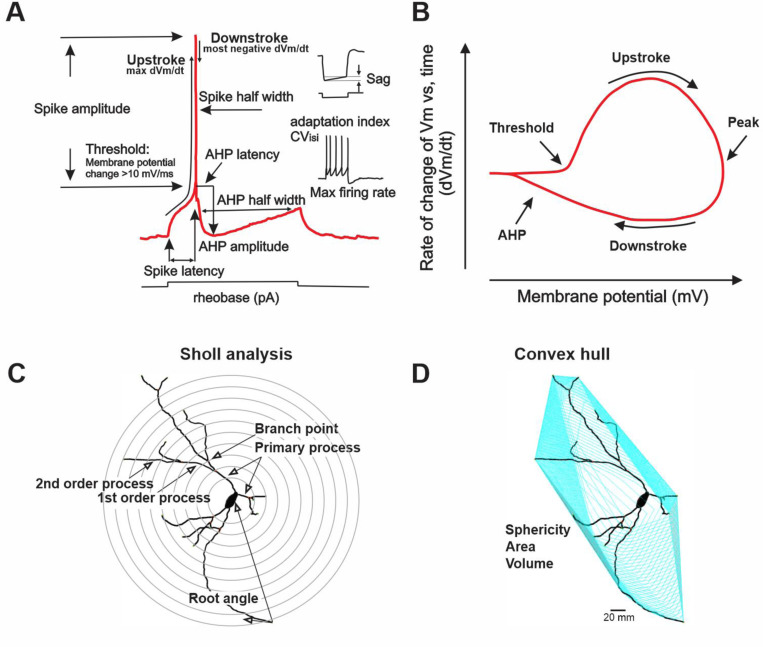
(related to Figs. 2, 4, 7 and 9) Physiological and morphological parameters used for morpho-electric profiling. **A**. Schematic of an action potential with all electrophysiological parameters assayed at rheobase noted. Max firing rate (inset) was measured in response to a 200 pA x 500 mS pulse with the rate calculated as the spikes during the 500 ms pulse x 2, Hz. Adaption index was calculated as the number of spikes in the first vs the second half of the 500 ms pulse. CV ISI was assayed as the coefficient of variation of the interspike interval over the entire 500 mS x 200 pA. 10 pA steps from - 60 pA hyperpolarizing pulses to + 200 pA were applied. **B**. Schematic of phase plot showing the membrane potential (Vm) rate of change vs time (dV_m_/dt) plotted vs membrane potential throughout the cycle of firing at rheobase. The basic features of a phase plot including upstroke, downstroke, etc are indicated. **C**. Schematic diagram of Sholl analysis and proximal dendritic parameters assayed following skeletonized representation of relocalized, neurobiotin stained neurons in Imaris. **D**. Illustration of convex hull analysis of proximal somatodendritic domain following skeletonized representation of relocalized, neurobiotin stained neurons in Imaris.

**Fig. 2–2:**
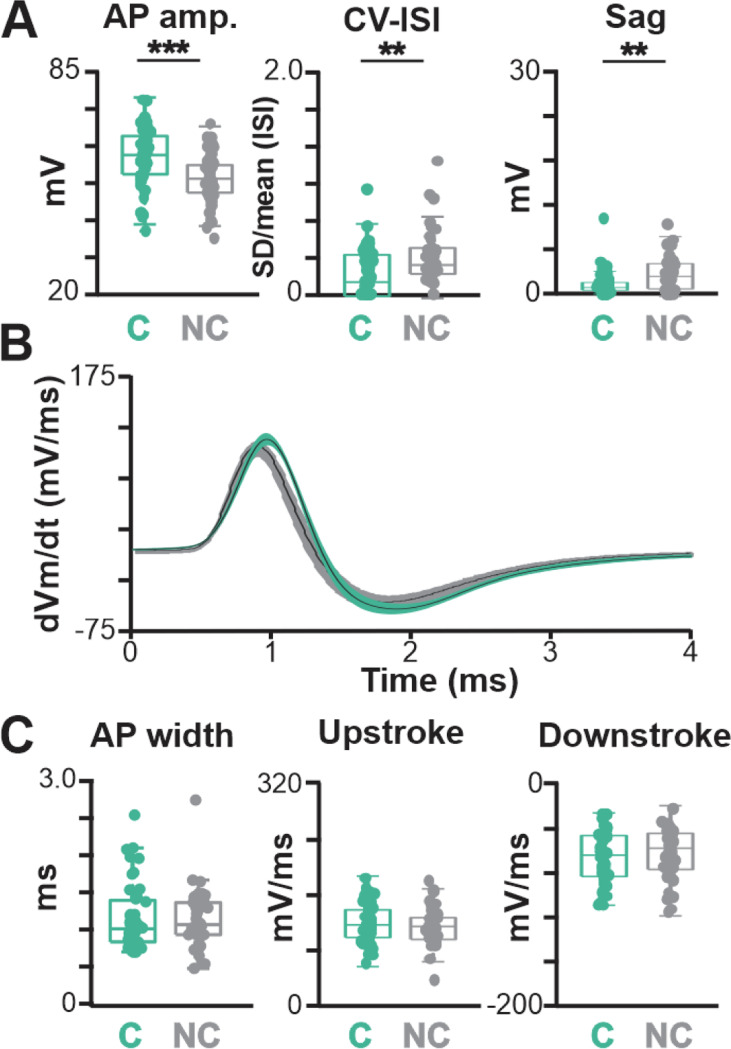
Additional electrophysiological features that distinguish BLA-projecting, cholinergic from noncholinergic neurons and features that are shared in mouse NBM/SI. **A**. Population scatter plus box plots of data for membrane properties of BLA-projecting, NBM/SI cholinergic neurons (n= 48) and their neighboring BLA-projecting, non-cholinergic neurons (n= 46; cholinergic: teal; non cholinergic: grey) **B**. Average time derivative vs time plots illustrate differences in action potential time course of BLA-projecting, NBM/SI neurons (cholinergic: teal; non cholinergic: grey). Shading represents SEM. **C**. Population scatter plus box plots of data for the 3 membrane features that are shared between BLA-projecting, NBM/SI cholinergic neurons and their neighboring BLA-projecting, non-cholinergic neurons (cholinergic: teal; non-cholinergic: grey) C, cholinergic; NC, non-cholinergic

**Fig. 3–1:**
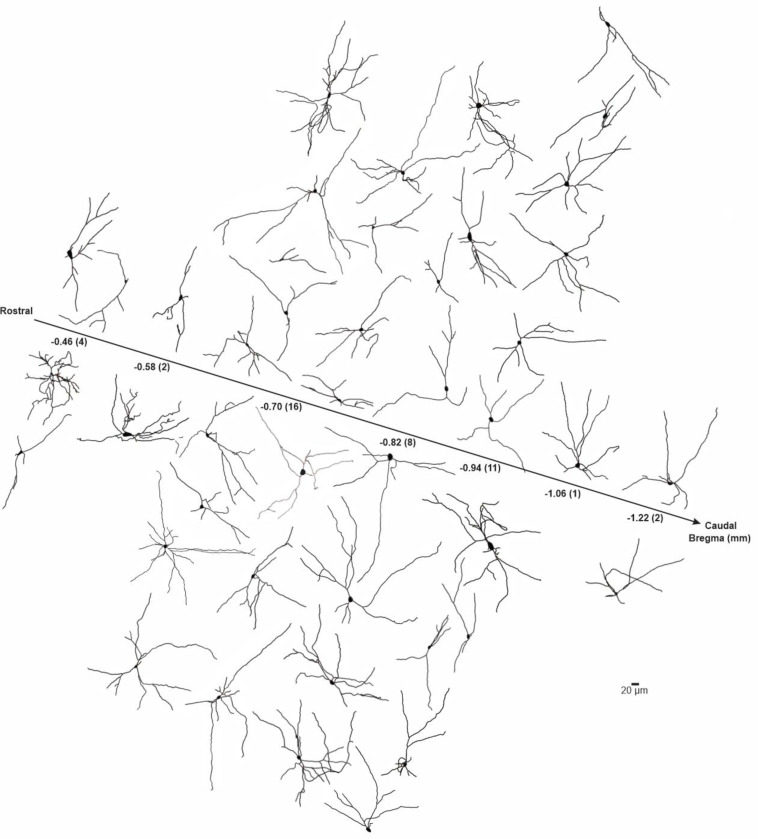
Skeletonized renditions of relocalized BLA-projecting non-cholinergic neurons within NBM/SI along bregma in mouse. Similar to cholinergic neurons, the proximal 100^+^ μm of the processes emanating from cholinergic somata were morphologically diverse regardless of location along bregma. All neurons were bi-polar or multi-polar.

**Fig. 4–1:**
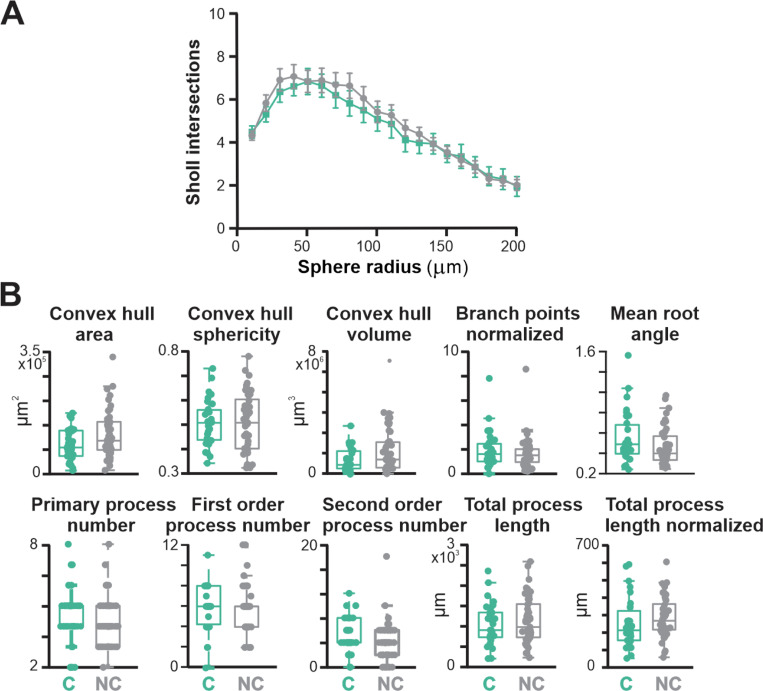
Most morphological features of somatodendritic domains are shared by neighboring NBM/SI neurons that project to the BLA for cholinergic and non-cholinergic in mouse. **A:** Sholl intersections analysis as a function of sphere radius from cell soma. **B:** Ten additional aspects of proximal neuritic morphology in neighboring NBM/SI neurons that project to the BLA. Majority of cell shape and proximal neurite configuration parameters are shared amongst BLA-projecting cholinergic and non-cholinergic NBM/SI neurons in mouse, despite marked differences in electrophysiological properties. C, cholinergic (n = 31); NC, non-cholinergic (n = 44)

**Fig. 6–1:**
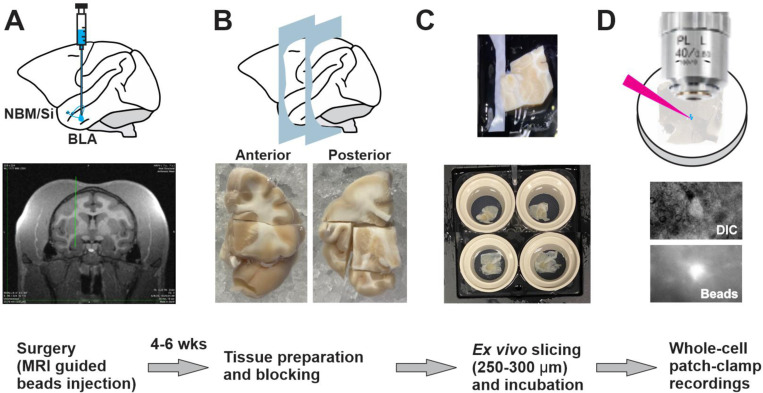
Workflow for preparation of macaque *ex vivo* recording. **A**. **Beads injection** Pre-operative structural MRIs were used to determine the stereotaxic injection coordinates for BLA fluorescently tagged microbeads injection. **B**. **Tissue blocking** About 4–6 weeks post op, a Br −4.0 to −8.0 section is surgically removed from ipsilateral macaque brain and is blocked into a small piece containing NBM/SI. **C**. **Slicing and incubation** The tissue block is glue to the vibratome platform and cut from medial to lateral. Slices are incubated at room temperature until being used for recording. **D**. ***Ex vivo* slice recording** Slices are examined under both DIC and fluorescent microscope for locating beads labeled neurons for recording.

**Fig. 6–2:**
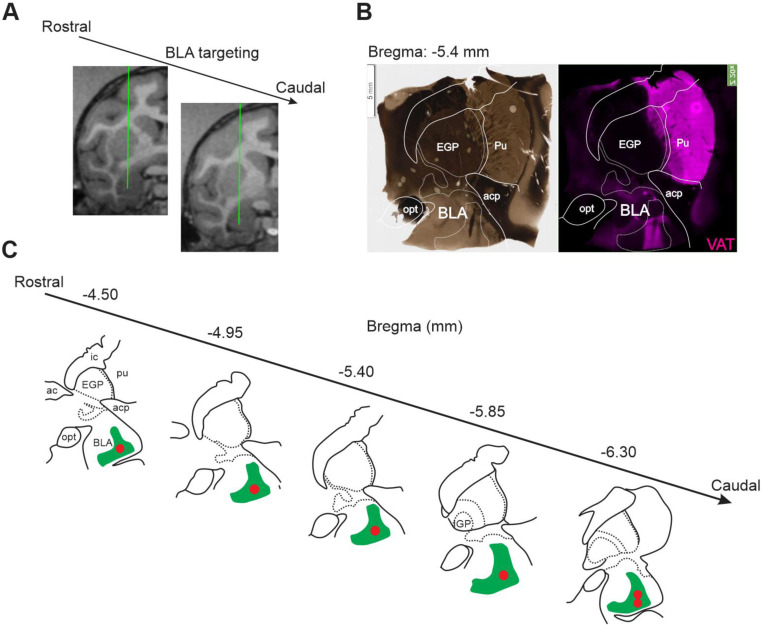
Reconstruction of the microsphere injection sites in macaque BLA for retrograde labeling of BLA-projecting neurons. **A:** MRI guided BLA beads injection in two coronal plates separated by 1.5 – 2.0 mm; green lines on each image indicate the injection tracks. Three sites within each track were targeted along the dorsoventral axis, spaced 2 – 2.5 mm apart. 20 μL was injected at each site, for a total volume of 120 μL across 6 sites. **B:** Left, a representative bright field slice image showing major landmarks and the beads injection site. Right, same slice, stained with VAT antibody, shows high cholinergic terminal density and beads colocalization within BLA. **C:** Beads target identification on slices in each animal.

**Fig.6–3:**
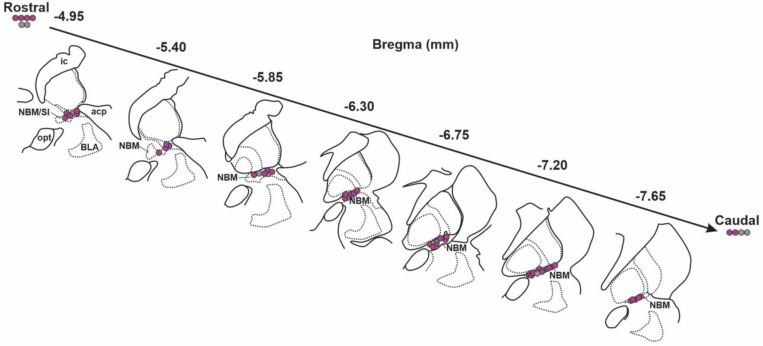
Approximate location of confirmed cholinergic BLA-projecting neurons that were subject to subsequent electrophysiological recording in rhesus macaque. Schematic diagram of coronal views of NBM/SI regions. Approximate locations of all confirmed BLA-projecting, NBM/SI cholinergic (ChAT+) neurons (n= 52) are shown in purple; the locations of BLA-projecting, ChAT negative neurons (n= 11) are shown in grey. The confirmed ChAT+ re-localized neurons comprise the sample for the electrophysiological and morphological features described.

**Fig.7–1:**
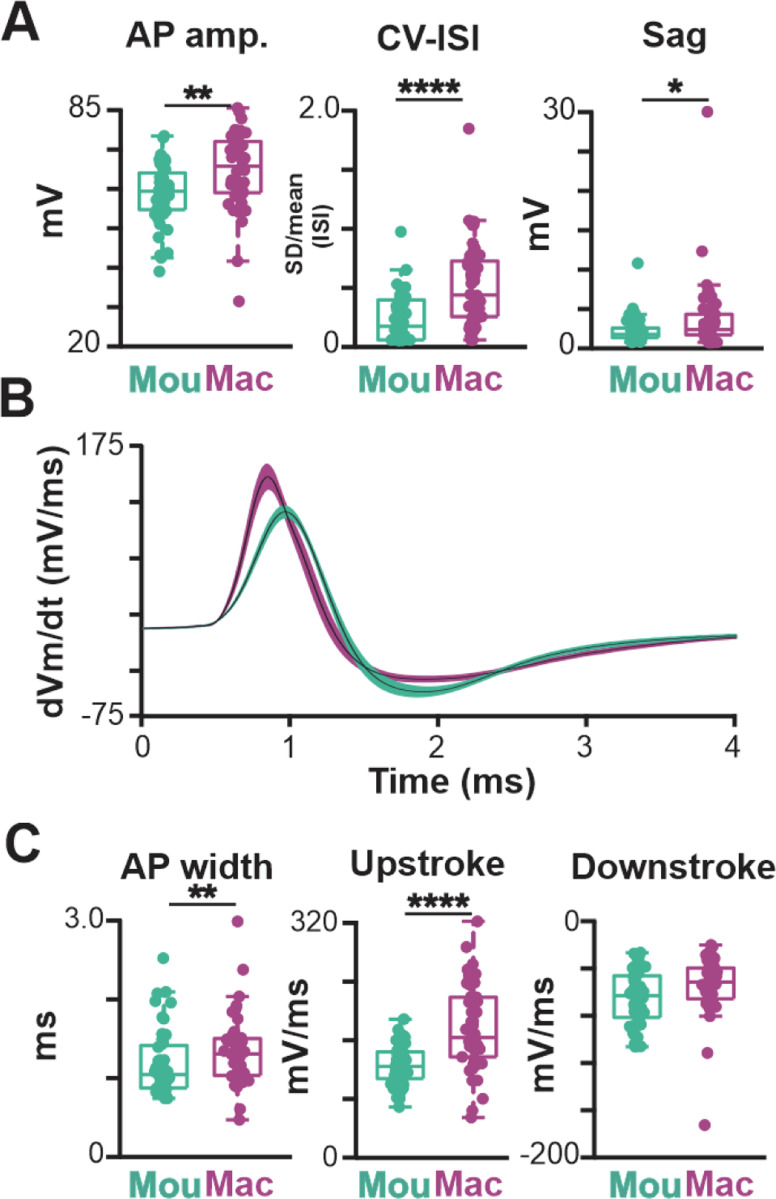
Additional electrophysiological features of BLA-projecting, cholinergic neurons in NBM/SI of rhesus macaque vs mouse. **A**. Population scatter plus box plots of additional features that differ in the action potential time course of BLA-projecting, NBM/SI cholinergic neurons from macaque (n= 46; purple) vs. BLA-projecting, cholinergic neurons in mouse (n= 48; teal). Mouse data are the same as those in Figs. 2 and 2-2, presented here for ease of comparison. **B**. Time derivative *vs* time plots illustrate differences in action potential time course of ChAT + BLA-projecting, NBM/SI neurons from macaque (purple) compared with mouse (teal). Mouse data are the same as those in Fig. 2-2 for ease of comparison. **C**. Population scatter plus box plots showing features of AP width and Upstroke are distinct, but feature of downstroke is shared between BLA-projecting, NBM/SI cholinergic neurons from macaque (purple; n= 46) and mouse (teal; n= 48). Mouse data are the same as those in Figs. 2 and 2-2, presented here for ease of comparison. p value symbols used are ** ≤ 0.01; unlabeled comparisons are not statistically significantly different.

**Fig. 9–1:**
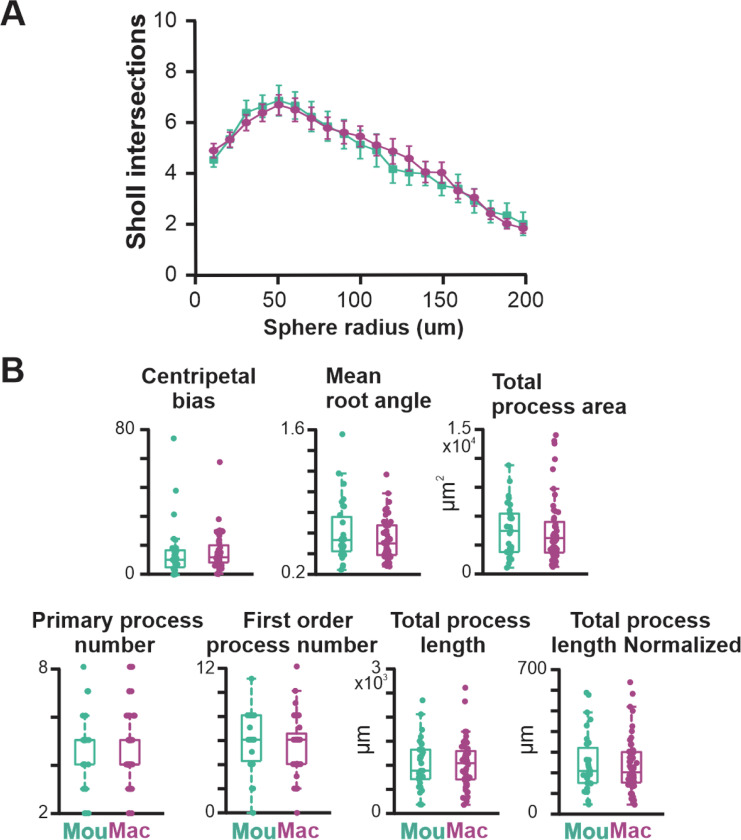
Additional morphological parameters assessed in BLA projecting, NBM/SI, cholinergic neurons of rhesus macaque compared with those in mouse. **A**. Sholl intersections analysis as a function of sphere radius from cell soma; **B**. Seven additional aspects of proximal somatodendritic morphology were assessed in NBM/SI, BLA projecting cholinergic, neurons from macaque (purple n=52) compared with those from mouse (n = 31, teal). Mouse data are from the same set of morphological parameters as presented in Figs. 4 and 4-1.

**Fig. 9–2:**
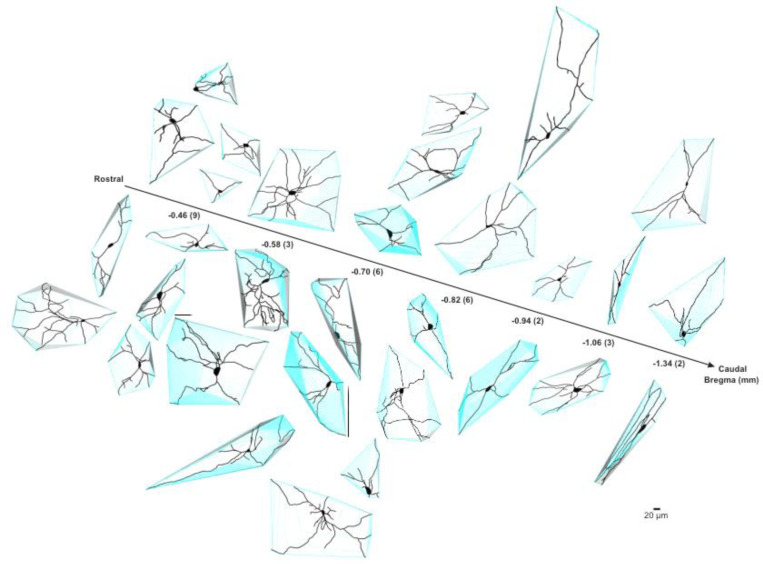
Reconstructed images of relocalized BLA-projecting cholinergic neurons within NBM/SI outlined by fitted convex hulls along bregma in mouse. Mouse neurons are smaller than macaque neurons in volume and occupy relatively small three-dimensional physical space.

**Fig. 9–3:**
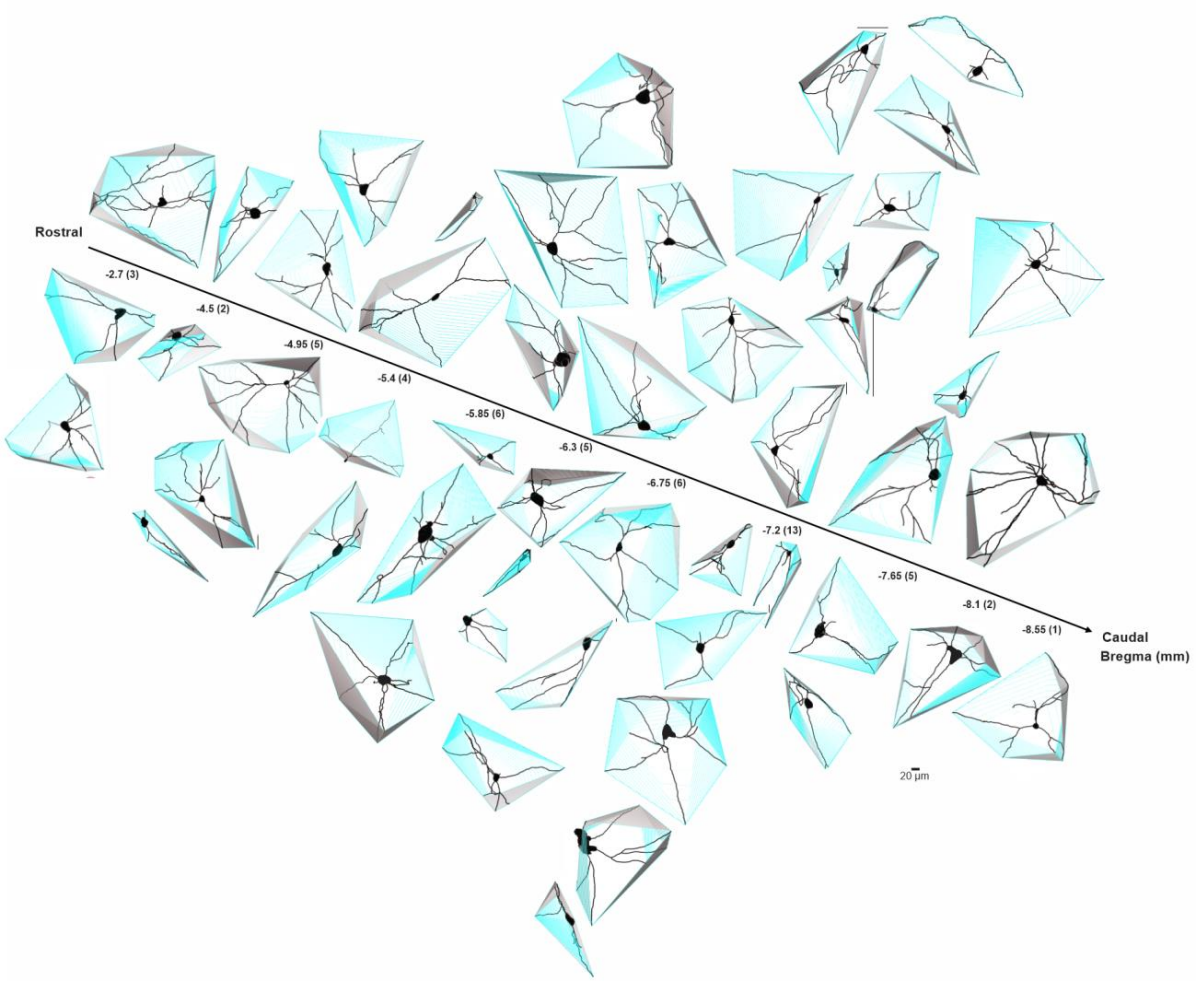
Reconstructed images of relocalized BLA-projecting cholinergic neurons within NBM/SI outlined by fitted convex hulls along bregma in macaque. In comparison to mouse neurons, macaque neurons are larger in volume and occupy more three-dimensional physical space.

## Figures and Tables

**Fig. 1:**
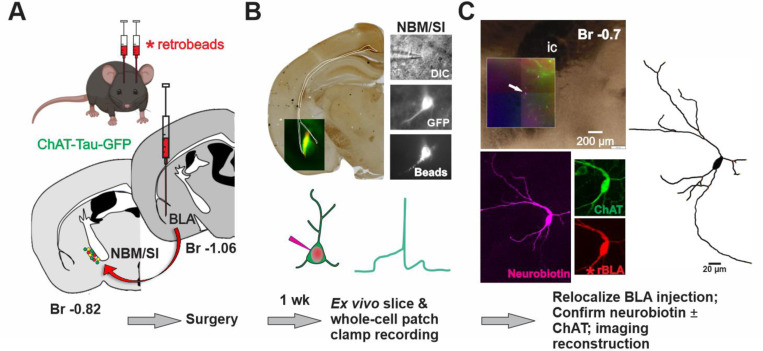
Workflow for morpho-electric profiling of NBM/SI BLA-projecting cholinergic and non-cholinergic neurons in mouse. See Extended Data Figs. 1-1 and 1-2. **A**. **Surgical back-labeling** Schematic for injection of fluorescently tagged microbeads into bilateral BLA of Chat-Tau-GFP mice (gift from S. Vijayaraghavan, Univ. Colorado, Grybko et al., 2011). Stereotactic delivery of 200~300 nl of red fluorescence tagged, *microbeads (FluoSpheres^™^ Carboxylate-Modified Microspheres, F8793, Invitrogen*) at approximately −1.1 mm bregma (L/M, +/− 3.25 mm & D/V −4.15 mm from dura) of a 6-week-old ChAT-tau-GFP mouse to retrograde-label BLA projecting neurons. (Below: RHS) schematic of coronal section illustrating approximate stereotactic positioning of injection pipette and ipsilateral back labeling of cholinergic (red + green, yellow) and non-cholinergic (red) neurons within the NBM/SI region examined (~−0.7– 0.9 *mm* Bregma). Green symbols represent Chat-Tau-GFP cell bodies that were not beads labeled. **B. Live imaging & recording** About 1 week post op, mice are prepared for *ex vivo*, slice recording. (top left) Photo micrograph of sample injection site of red beads into the BLA of a ChAT-tau-GFP mouse. Yellow and green signal in BLA derives from the extensive (green fluorescent) cholinergic terminal fields within this area. (Top right) photomicrographs taken during the recording session in the region of the NBM/SI. Cells were first identified in DIC (top); ChAT-tau-GFP labeling was readily detected in live imaging of NBM/SI cholinergic neurons. BLA-projecting neurons were readily detectable in live imaging due to beads from the BLA injection. If the neuron was labeled with both ChAT GFP and the red beads it was classified as BLA projecting & cholinergic. BLA projecting, non-cholinergic neurons are defined as bead labeled, without ChAT-Tau-GFP label. All neurons were injected with Neurobiotin (SP-1120, Vector Laboratories) during electrophysiological recording. **C. Relocalization** Neurons were relocalized based on coordinates and Neurobiotin staining with streptavidin, ChAT and/or beads labeling. Confirmed images were imported into Imaris for morphological parameter assessment.

**Fig. 2:**
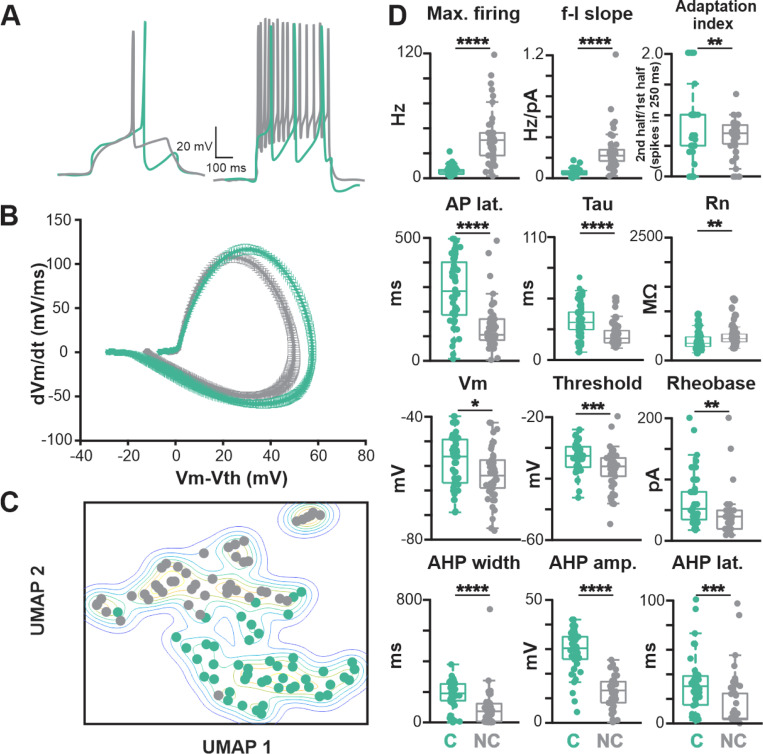
Electrophysiological features consistent with the lower excitability of mouse cholinergic BLA-projecting NBM/SI neurons compared with neighboring non-cholinergic BLA-projecting NBM/SI neurons. See Extended Data Figs. 2-1 and 2-2. **A**. Sample traces at rheobase (left) and at maximum current injection (right; 200 pA) are shown for typical BLA-projecting, NBM/SI neurons (cholinergic: teal; non cholinergic: grey) **B**. Average phase plots illustrate differences in action potential kinetics comparing BLA-projecting, NBM/SI neurons (cholinergic: teal; non cholinergic: grey) **C**. Non dimensional (UMAP) plot of all 18 electrophysiological features comparing BLA-projecting, NBM/SI neurons (cholinergic: teal; non cholinergic: grey) **D**. Population scatter plus box plots of all data for the 12 features that most strongly distinguish BLA-projecting, NBM/SI cholinergic neurons (n = 48) from their neighboring BLA-projecting, non-cholinergic neurons (n = 46; cholinergic: teal; non cholinergic: grey) p value symbols used in this and all subsequent figures are * ≤0.05; ** ≤ 0.01; *** ≤ 0.001; **** ≤ 0.0001. C, cholinergic (n = 48); NC, non-cholinergic (n = 46)

**Fig.3:**
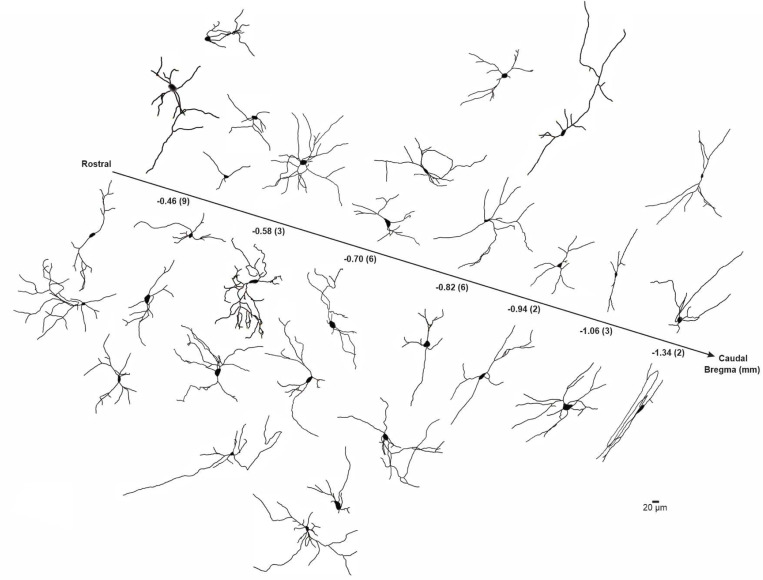
Skeletonized renditions of relocalized BLA-projecting cholinergic neurons within NBM/SI along all bregma in mouse. See Extended Data Fig. 3-1. The proximal 100^+^ μm of the processes emanating from cholinergic somata were morphologically diverse and independent of location along bregma. Most neurons were multi-polar although fairly simple in morphology (n = 31).

**Fig.4:**
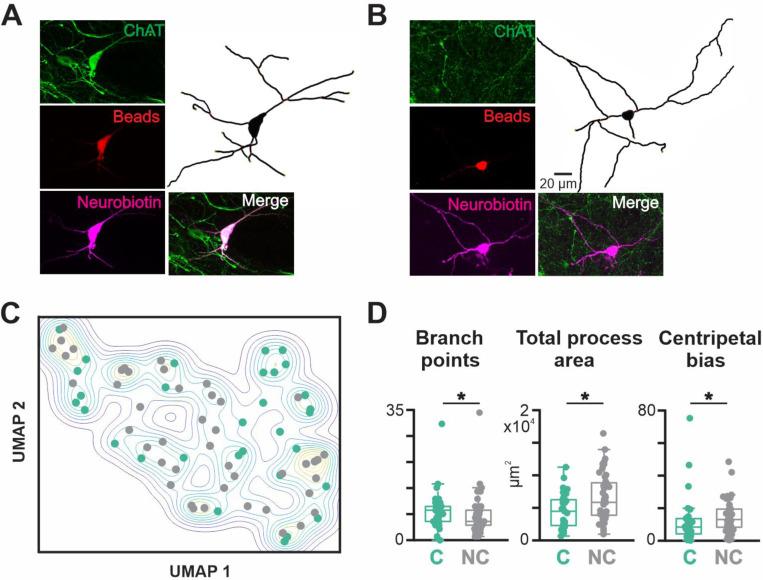
BLA-projecting, NBM/SI neurons in mouse, whether cholinergic or noncholinergic, differ in 3 of the 13 morphological parameters assessed. See Extended Data Fig. 4-1. **A**. Confocal images of a representative BLA-projecting cholinergic neuron in mouse **B**. Confocal images of a representative BLA-projecting non-cholinergic neuron in mouse **C**. Non-dimensional (UMAP) plot of the morphological features of BLA-projecting, NBM/SI neurons (cholinergic: teal; non cholinergic: grey) **D**. Population scatter plus box plots of data for the 3 morphological features that are differed significantly between BLA-projecting, NBM/SI cholinergic neurons (n = 31) and their neighboring BLA-projecting, non-cholinergic neurons (n = 44; cholinergic: teal; non cholinergic: grey) “C” stands for cholinergic, and “NC” stands for non-cholinergic.

**Fig.5:**
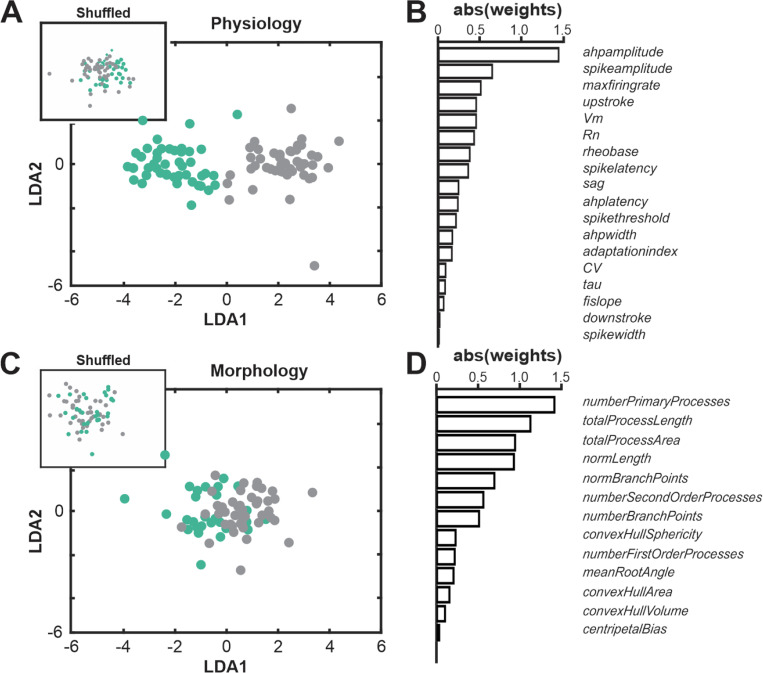
Linear Discriminant Analysis (LDA) strongly distinguishes cholinergic from non-cholinergic BLA projecting NBM/SI mouse neurons based on electrophysiological, but not on morphological features. **A**. Linear discriminant analysis was applied to all electrophysiological features for BLA-projecting, NBM/SI neurons (cholinergic: teal, n = 48; non cholinergic: grey, n = 46). There is clear separation in clustering of the two populations from one another but not from the distribution of shuffled data (shown in inset), consistent with the many electrophysiological features that distinguish between BLA-projecting cholinergic vs. non-cholinergic neurons in mouse. **B**. Plot of the absolute values of the weighted differences in electrophysiological features between BLA-projecting, NBM/SI neurons (cholinergic: teal; non cholinergic: grey). **C**. Linear discriminant analysis was applied to all morphological features for BLA-projecting, NBM/SI neurons (cholinergic: teal, n = 31; non cholinergic: grey, n = 44). The two populations don’t separate from one another nor is the distribution of morphological features very different from the shuffled data (shown in inset). **D**. Plot of the absolute values of the weighted differences in morphological features between BLA-projecting, NBM/SI neurons (cholinergic: teal; non cholinergic: grey) of mouse.

**Fig. 6:**
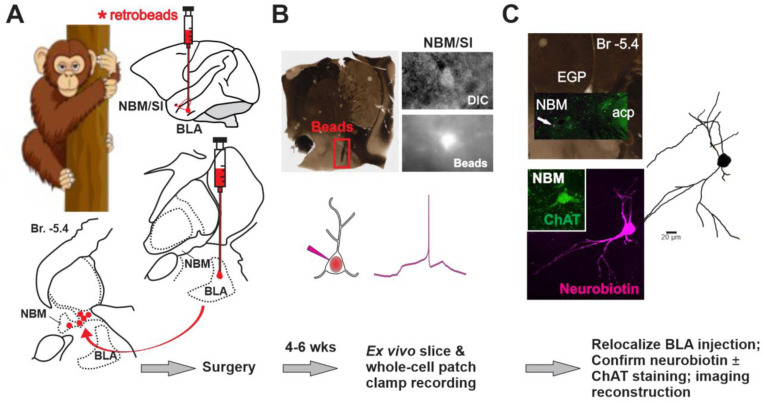
Workflow for morpho-electric profiling of BLA-projecting NBM/SI cholinergic neurons in macaque. See Extended Data Figs. 6-1, 6-2 and 6-3. **A**. **Surgical retrograde-labeling** (top) Schematic for fluorescently tagged microbeads injection into the basolateral amygdala (BLA) for retrograde labeling of BLA projecting basal forebrain neurons in rhesus macaque. MRI guided and stereotactic delivery of 120 μl of fluorescence tagged *microbeads* into the region of the BLA (Bregma −6.75 to −7.75 mm at approx. L/M 7.5 mm x D/V 34.0 mm in either right or left hemisphere) of ~7 – 13-year-old rhesus macaques to back-label BLA projecting neurons (see supplementary for more details). (Below: RHS) Schematic of coronal section illustrating approximate track and positioning of bead injection needle. (Bottom: LHS) Schematic of coronal view to illustrate target of ipsilateral retrograde labeling within the NBM/SI region examined (~−5.4 mm Bregma). **B. Live imaging & recording:** About 4–6 weeks post op, a Br −4.0 to −8.0 mm section is surgically removed from side ipsilateral to the beads injection of the macaque brain and is prepared for *ex vivo*, slice recording (Top left and see Fig. 6-1B). Top right: Photomicrographs taken during the recording session in the region of the NBM SI, Cells were identified in DIC (top) and BLA-projecting neurons detected in live imaging by microbeads from the BLA injection. Bead positive neurons were injected with Neurobiotin tracer during electrophysiological recording. **C. Relocalization:** Neurons were relocalized based on coordinates and neurobiotin staining. ChAT labeling was confirmed by immunostaining and high-power images of the cell body and proximal dendrites were imported into Imaris for morphological parameter assessment.

**Fig. 7:**
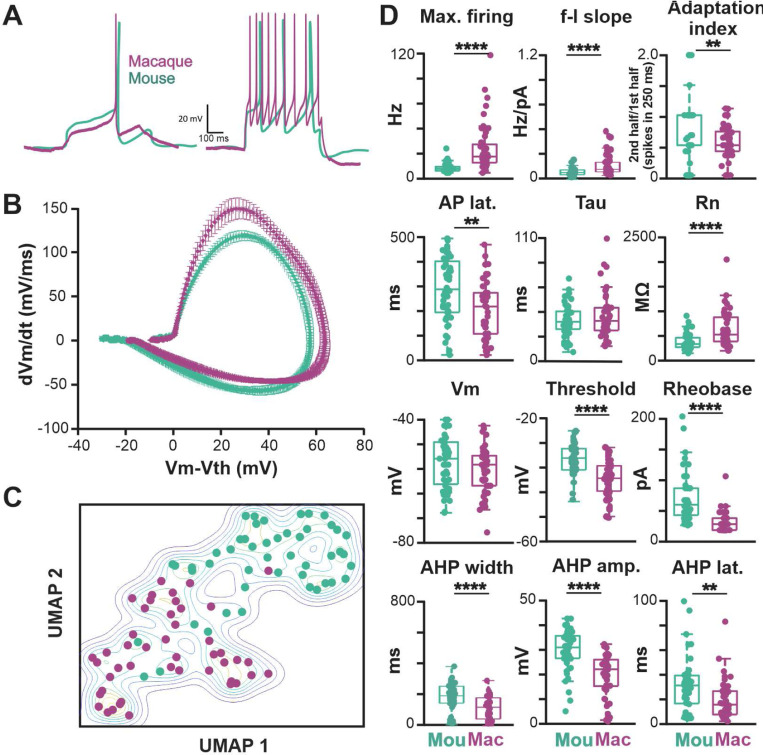
Both passive and active membrane properties of macaque BLA-projecting, NBM/SI cholinergic neurons are consistent with higher excitability than those of mouse. See Extended Data Fig. 7-1. **A**. Sample traces from BLA projecting, cholinergic NBM/ SI macaque neurons at rheobase (left) and at maximum current injection (right; 200 pA) are shown (macaque: purple; mouse: teal) Mouse data (from same data base of BLA-projecting cholinergic neurons as in Fig. 2A) are shown here again for comparison purposes. **B**. Average phase plots illustrate differences in action potential kinetics between macaque, BLA-projecting, NBM/SI cholinergic neurons (purple) and mouse (teal); Mouse data are the same as those in Fig. 2B, presented here for comparison purpose. **C**. Non dimensional (UMAP) plot of all 18 electrophysiological features comparing BLA-projecting, NBM/SI cholinergic neurons from macaque (purple) with comparable samples from mouse (teal). Mouse data are the same as those in Fig. 2C, presented here for ease of comparison. **D**. Population scatter plus box plots of data for 12 features that distinguish BLA-projecting, NBM/SI cholinergic neurons of the rhesus macaque (n = 46; purple) from BLA-projecting, NBM/SI cholinergic neurons from mouse (n = 48; teal). Mouse data are the same as those in Fig. 2D presented here for ease of comparison.

**Fig.8:**
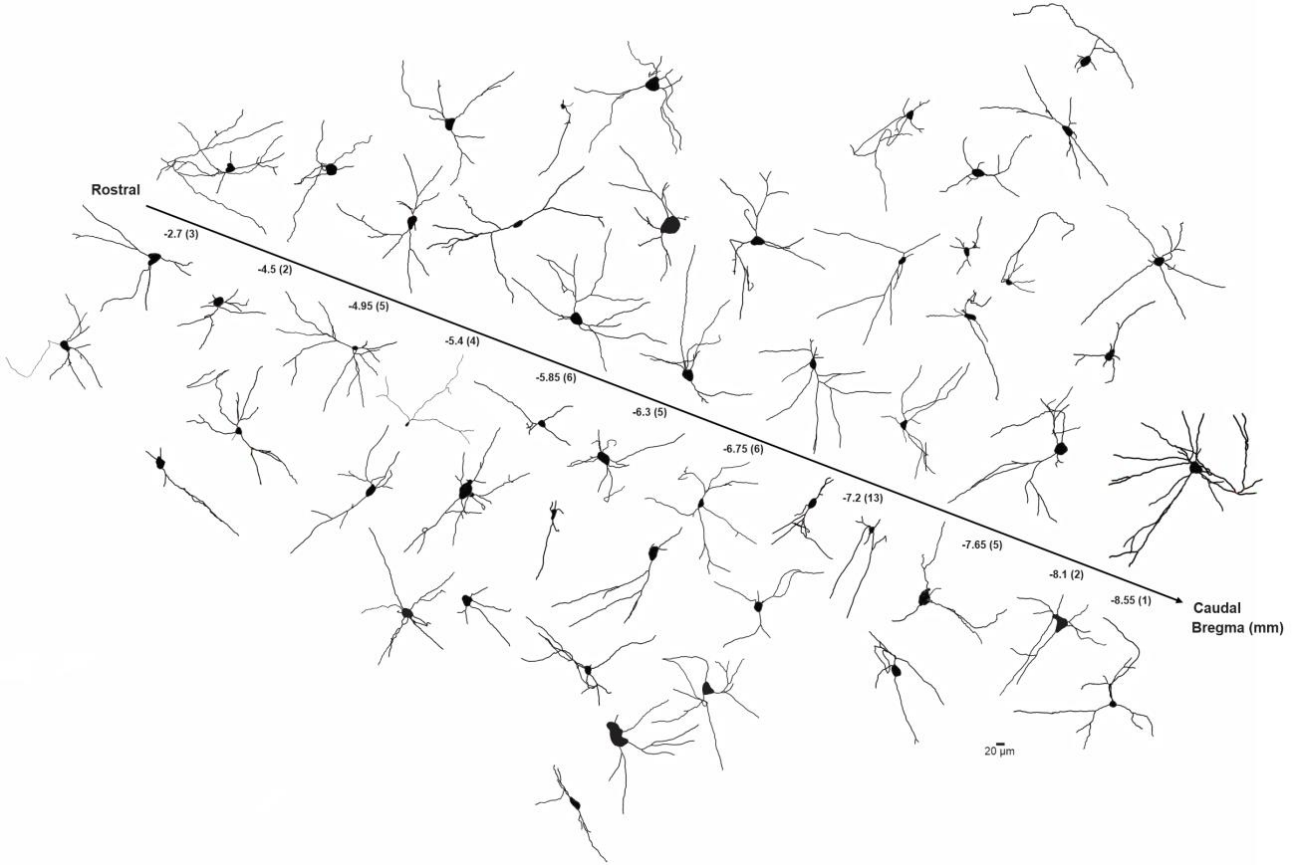
Skeletonized renditions of relocalized BLA-projecting cholinergic neurons within NBM/SI along bregma in macaque. The proximal 100^+^ μm of the processes emanating from cholinergic somata of macaque were morphologically diverse regardless of location along bregma. Most neurons were multi-polar, yet fairly simple (n = 52).

**Fig. 9:**
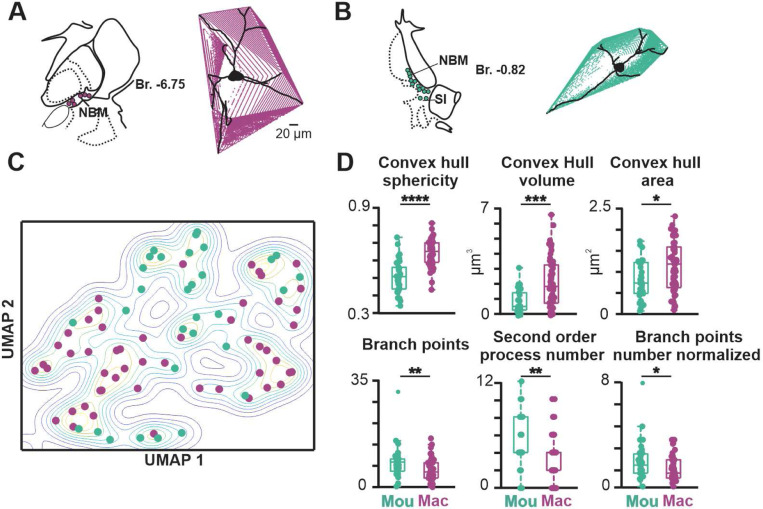
BLA projecting, NBM/SI, cholinergic neurons in macaque differ from those in mouse in 6 of the 13 morphological parameters assessed. See Extended Data Figs. 9-1, 9-2 and 9-3. **A**. (LHS) Approximate location in hemi-coronal diagram of macaque basal forebrain region at ~−6.75 Brega. (RHS) A volume rendering of proximal neuritic domain of a representative NBM/SI, BLA projecting, cholinergic neuron from macaque **B**. Approximate location in hemi coronal section of mouse basal forebrain and volume rendering of proximal neurite domain of a representative NBM/SI, BLA projecting, cholinergic neuron from mouse **C**. Non dimensional (UMAP) plot of all 13 morphological features of BLA-projecting, NBM/SI, cholinergic neurons from macaque (purple, n = 52) vs mouse (teal, n = 31). Mouse data are from the same set of morphological parameters as presented in Figs. 4 and 4-1. **D**. Population scatter plus box plots of data for the 6 set of morphological properties that differ between BLA-projecting, NBM/SI cholinergic macaque (n = 52; purple) compared with mouse (n = 31; teal) neurons. Mouse data are from the same set of morphological parameters as presented in Figs. 4 and 4-1.

**Fig. 10:**
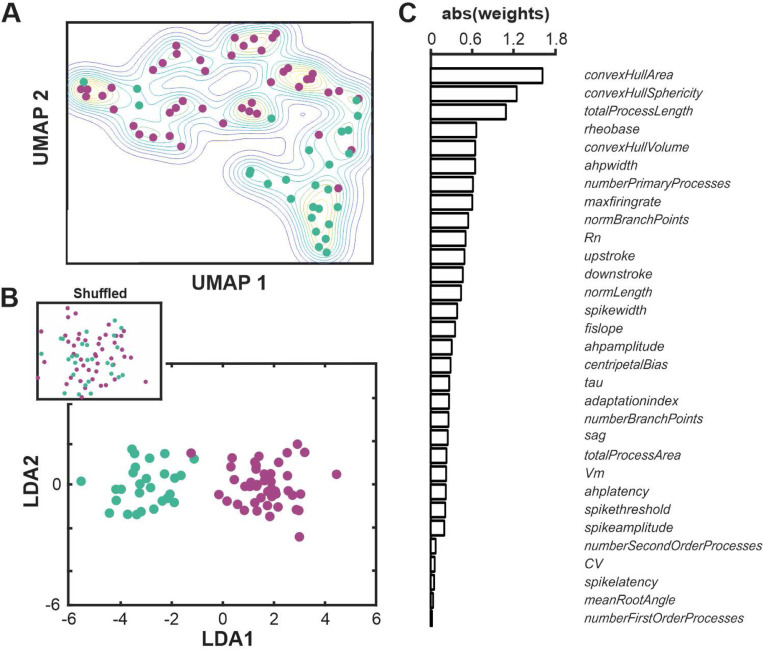
Examination of combined morpho-electric features distinguish macaque vs mouse basal forebrain neurons despite common projection target, anatomical location, and cholinergic phenotype. **A**. Non dimensional (UMAP) plot of all 18 electrophysiological and 13 morphological features of BLA-projecting, NBM/SI, cholinergic neurons from macaque (purple, n = 46) vs mouse (teal, n = 27). **B**. Linear discriminant analysis was applied to all 18 electrophysiological and 13 morphological features of BLA-projecting, NBM/SI, cholinergic neurons from macaque (purple, n = 46) vs mouse (teal, n = 27). Note the clear separation in clustering of the two populations from one another and its distinct nature from the distribution of shuffled data (shown in inset). **C**. Plot of the absolute values of the weighted differences for LDA in all 18 electrophysiological and 13 morphological features of BLA-projecting, NBM/SI, cholinergic neurons from macaque (purple, n = 46) vs mouse (teal, n = 27).

**Table 1:** Summary of electrophysiological data collected from chat-tau-GFP mouse and macaque with BLA microbeads injection.

species	N	Sex	Age	BLA-proj. chol., n	BLA-proj. non-chol., n
mouse	27	18M; 9F	5–14 wks (Median 7 wks)	48	46
macaque	9	7M; 2F	7–17 yrs (Median 10 yrs)	46	11

## SIGNIFICANCE STATEMENT

Cholinergic neurons of the basal forebrain participate in memory functions essential for survival and for providing emotional context to otherwise neutral cognitive stimuli (Picciotto et al., 2012; Ballinger et al., 2016; Ananth et al., 2023). Here we assess, in detail, the electrophysiological and morphological properties of a specific subset of basal forebrain cholinergic neurons (BFCNs) in mouse and in rhesus macaque. By focusing our comparison on BLA-projecting NBM/SI cholinergic neurons that are engaged, in both species, in vital and lifelong aspects of memory encoding, we provide quantitative data that establish important boundary conditions for how knowledge of cholinergic structure and function (at the cellular level) obtained in the rodent may be transferred to an understanding of the primate.
